# Fovea-like Photoreceptor Specializations Underlie Single UV Cone Driven Prey-Capture Behavior in Zebrafish

**DOI:** 10.1016/j.neuron.2020.04.021

**Published:** 2020-07-22

**Authors:** Takeshi Yoshimatsu, Cornelius Schröder, Noora E. Nevala, Philipp Berens, Tom Baden

**Affiliations:** 1 School of Life Sciences, University of Sussex, Brighton BN1 9QG, UK; 2 Institute of Ophthalmic Research, University of Tübingen, Tübingen 72076, Germany; 3 Center for Integrative Neuroscience, University of Tübingen, Tübingen 72076, Germany; 4 Institute for Bioinformatics and Medical Informatics, University of Tübingen, Tübingen 72076, Germany

**Keywords:** cone, photoreceptor, fovea, retina, zebrafish, prey capture, phototransduction, UV vision, ribbon synapse

## Abstract

In the eye, the function of same-type photoreceptors must be regionally adjusted to process a highly asymmetrical natural visual world. Here, we show that UV cones in the larval zebrafish area temporalis are specifically tuned for UV-bright prey capture in their upper frontal visual field, which may use the signal from a single cone at a time. For this, UV-photon detection probability is regionally boosted more than 10-fold. Next, *in vivo* two-photon imaging, transcriptomics, and computational modeling reveal that these cones use an elevated baseline of synaptic calcium to facilitate the encoding of bright objects, which in turn results from expressional tuning of phototransduction genes. Moreover, the light-driven synaptic calcium signal is regionally slowed by interactions with horizontal cells and later accentuated at the level of glutamate release driving retinal networks. These regional differences tally with variations between peripheral and foveal cones in primates and hint at a common mechanistic origin.

## Highlights

• Larval zebrafish prey detection uses specialized single UV cones in the acute zone
• High-gain UV cones in this region boost detection of UV-bright contrasts
• Cellular and molecular mechanisms of this tuning tally with those of the primate fovea
• Further optimization occurs at the level of UV cone glutamate release

## Graphical Abstract

**Figure:**
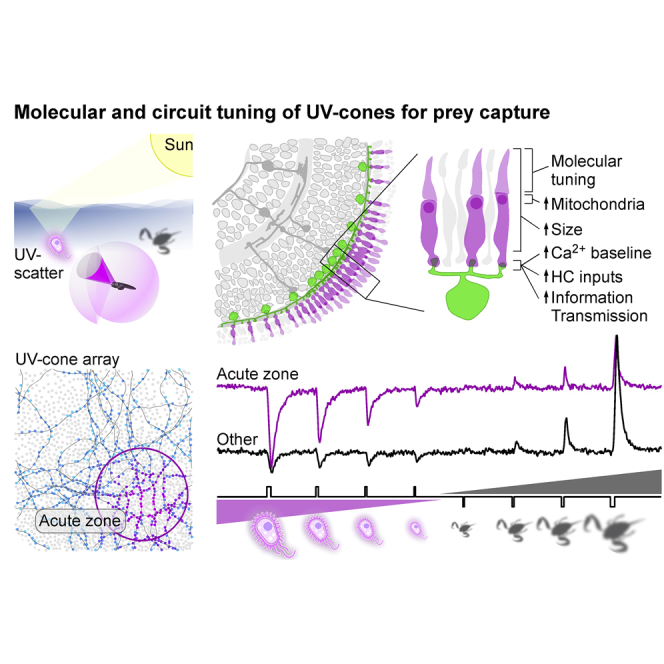

## Teaser

Yoshimatsu et al. show that larval zebrafish rely on single UV cones at a time to support visual prey capture. For this, zebrafish combine molecular, cellular, and circuit tuning to regionally boost detectability of prey in their acute zone. The mechanisms of this specialization tally with those of the primate fovea.

## Introduction

In vision, photoreceptors drive the retinal network through continuous modulations in synaptic release (Baden et al., 2013a, Heidelberger et al., 2005, Lagnado and Schmitz, 2015, Moser et al., 2020, Regus-Leidig and Brandstätter, 2012, Thoreson, 2007). However, how changes in incoming photon flux lead to changes in the rate of vesicle fusion at the synapse varies dramatically between photoreceptor designs (Bellono et al., 2018, Sterling and Matthews, 2005, Thoreson, 2007). For example, in the vertebrate retina, the slow rod photoreceptors typically have large outer segments and high-gain intracellular signaling cascades to deliver single-photon sensitivity critical for vision at low light (Field et al., 2005, Lamb, 2016, Yau and Hardie, 2009). In contrast, cone photoreceptors are faster and have smaller outer segments and lower-gain cascades to take over where rods saturate (Lamb, 2016, Yau and Hardie, 2009). Clearly, matching the properties of a given photoreceptor type to a specific set of sensory tasks critically underpins vision. However, these visual requirements can differ dramatically across the retinal surface and the corresponding position in visual space (Baden et al., 2013b, Hardie, 1984, Land and Nilsson, 2012, Sancer et al., 2019, Yilmaz and Meister, 2013, Zimmermann et al., 2018). For efficient sampling (Cronin et al., 2014, Land and Nilsson, 2012), even cones of a single type must therefore be functionally tuned depending on their retinal location.

Indeed, photoreceptor tuning, even within type, is a fundamental property of vision in both invertebrates (Hardie, 1984, Sancer et al., 2019) and vertebrates (Baden et al., 2013b, Baudin et al., 2019, Sinha et al., 2017). Even primates make use of this trick; foveal cones have longer integration times than their peripheral counterparts, likely to boost their signal to noise ratio, as in the foveal center, retinal ganglion cells (RGCs) do not spatially pool their inputs (Baudin et al., 2019, Sinha et al., 2017). Understanding the mechanisms that underlie such functional tuning will be important for understanding how sensory systems can operate in the natural sensory world and how they might have evolved to suit new sensory-ecological niches (Cronin et al., 2014, Lamb et al., 2007, Land and Nilsson, 2012, Yau and Hardie, 2009).

Here, we show that UV cones in the *area temporalis* (Schmitt and Dowling, 1999) (“strike zone” [SZ]; Zimmermann et al., 2018) of larval zebrafish are selectively tuned to detect microorganisms that these animals feed on (e.g., paramecia) (Westerfield, 2000, Spence et al., 2008).

## Results

### Larval Zebrafish Prey Capture Must Use UV Vision

**Figure 1:**
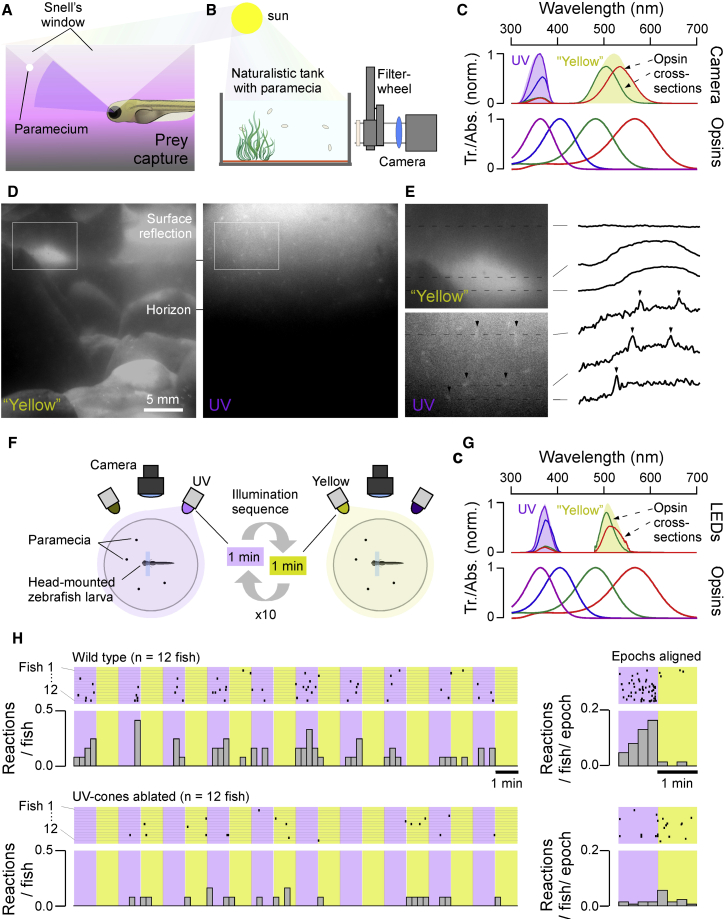
UV Light Greatly Facilitates Visually Guided Prey Capture in Larval Zebrafish (A) Schematic representation of visual prey capture by larval zebrafish. (B) Setup for filming paramecia. A filter wheel equipped with UV and yellow bandpass filters was positioned in front of the charge-coupled device (CCD) camera to image paramecia in a naturalistic tank in the sun. (C) Peak-normalized spectra for the UV and yellow channels (thick lines; STAR Methods) superimposed on the zebrafish’s four opsin absorption spectra (shadings). The spectral overlap between the UV and yellow channels with each opsin is indicated (thin lines). Abs., absorption; Tr., transmittance. (D) Example frames from the yellow and UV channels taken consecutively from the same position. (E) Zoom in from (D), with line profiles extracted as indicated. Arrowheads highlight paramecia visible in the UV channel. See also Video S1. (F) Schematic of behavioral setup. Individual larval zebrafish (7–8 dpf) in the presence of free-swimming paramecia were head-mounted and filmed from above, with infrared illumination from below. (G) Top illumination was provided by intensity-matched UV (374 ± 15 nm) or yellow (507 ± 10 nm) LEDs, which mainly activated UV/blue and red/green opsins, respectively, as indicated. (H) Top: zebrafish consistently responded more readily to passing paramecia with full prey-capture bouts (eye convergence + tail flicks, each event indicated with a marker) during UV-illumination periods. See also Video S2. Individual trials (left) and summary statistics (right). This difference was abolished when UV cones were ablated (bottom). Mann-Whitney *U* test, UV versus yellow light in wild-type (WT) fish: p < 0.01; WT versus UV killing under UV light: p < 0.001; UV versus yellow light in UV killing fish: p > 0.05; n = 12 each for WT and UV cone ablation.

Larval zebrafish prey capture is elicited by a bright spot of light (Bianco et al., 2011, Semmelhack et al., 2014), in line with the natural appearance of their prey items (e.g., paramecia) in the upper water column of shallow water when illuminated by the sun (Zimmermann et al., 2018; Figure 1A). To the human observer with comparatively long-wavelength vision (Nathans, 1999), these organisms are largely transparent when viewed against a back light (Johnsen and Widder, 2001). However, previous work suggests that zooplankton like paramecia scatter light in the UV band (320–390 nm) and thus appear as UV-bright spots (Novales Flamarique, 2012, Novales Flamarique, 2016, Zimmermann et al., 2018).

To explicitly test this idea, we custom-built a camera system with a UV and a “yellow” channel aligned with the zebrafish UV- and red/green-opsin absorption spectra, respectively (Chinen et al., 2003). We used this system to film free-swimming paramecia in a naturalistic tank placed outdoors under the midday sun (Figures 1B–1E; Video S1; STAR Methods). While the yellow image provided good spatial detail of the scene’s background and surface water movements, paramecia were difficult to detect among the background clutter (Figure 1D, left). In contrast, the UV channel was dominated by a vertical brightness gradient of scattered light, which almost completely masked the background. Superimposed on this gradient, the upper water column readily highlighted individual paramecia as bright moving spots (Figure 1D, right, and 1E). In agreement, zebrafish use their upper-frontal visual field to detect and capture prey (Bianco et al., 2011, Mearns et al., 2020, Patterson et al., 2013), and inner retinal circuits that process this part of visual space exhibit a strong, regionally specific bias for UV-bright contrasts (Zhou et al., 2020, Zimmermann et al., 2018). This confirmed that vastly different, and largely nonoverlapping types of information (Cronin and Bok, 2016) are obtainable from these two wavebands available to the zebrafish larvae. Any differences between the UV and yellow waveband (Figures 1D and 1E; Video S1) are likely to be further exacerbated by the fish’s self-movements relative to the scene. These would add major brightness transitions in the yellow, but not the UV, channel. Accordingly, under natural (rather than laboratory-controlled) viewing conditions, paramecia are likely hard to detect in the yellow waveband but readily stand out in the UV. This strongly suggests that larval zebrafish must capitalize on UV vision rather than achromatic or long-wavelength vision to support visual prey detection in nature (Cronin and Bok, 2016, Novales Flamarique, 2016, Zimmermann et al., 2018)

Video S1 Detecting Paramecia in UV and “Yellow” Wavebands, Related to Figure 1 Video of paramecia in naturalistic tank as viewed in a “yellow” channel that is approximately aligned with zebrafish M- and L-cones (left), and the same scene subsequently filmed in a zebrafish-approximate UV channel (right). The yellow channel provides spatial detail of the background and underside of the water, which masks paramecia swimming in the foreground. In contrast, the UV channel does not resolve the background clutter but instead brings out paramecia illuminated by the sun as bright dots in the upper water column. Videos recorded at 10 Hz and played back in real time (STAR Methods).

Indeed, UV illumination strongly facilitated behavioral performance: Head-mounted 7–8 days post-fertilization (dpf) larvae in the presence of free-swimming paramecia exhibited significantly more prey-capture attempts when illuminated with UV light (374 nm) compared to yellow light (507 nm) (Figures 1F–1H). This difference was abolished after genetic ablation of UV cones (Figure 1H, bottom; STAR Methods). Together, these results strongly suggest that UV cones provide the dominant input to visual prey-capture circuits in larval zebrafish.

#### Single UV Cones May Signal the Presence of Prey

**Figure 2:**
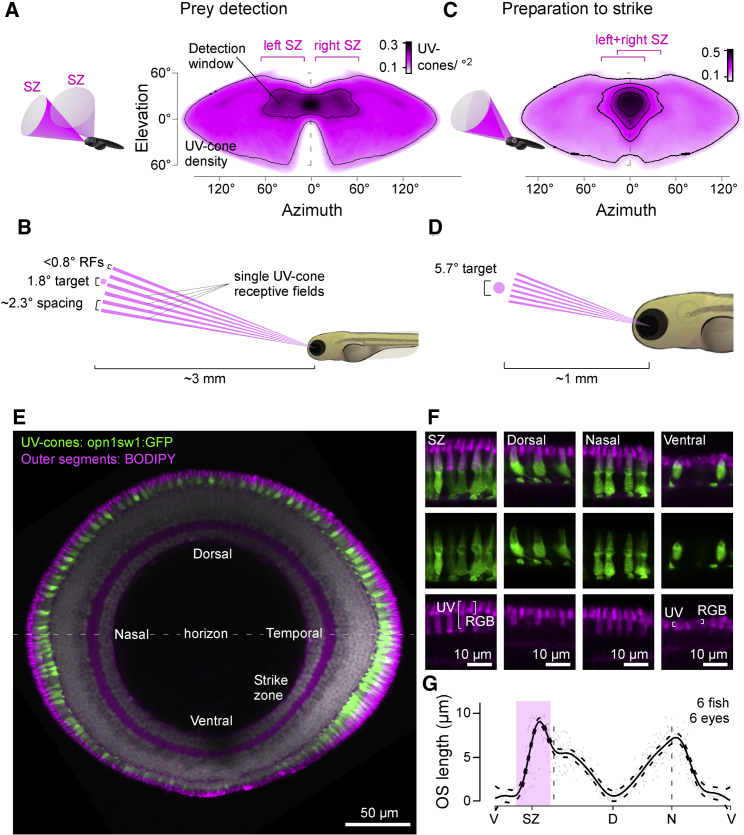
The Detector Hardware for UV Vision in Larval Zebrafish (A–D) UV cone density projected into sinusoidal map of visual space when eyes are in resting position for initial prey detection (A) and once converged for prey localization following detection (C). A 100-μm paramecium is too small for reliable detection at ∼3 mm distance and can therefore only be seen by a single UV cone at a time (B). Even at ∼1 mm strike distance, it covers at most a handful of UV cones per eye (D). 3D schematics (A and C) illustrate approximate visual space surveyed by the two SZs. Scale bars, UV cones/°. See also Figures S1A–S1D. (E) Sagittal section across the eye with outer segments (OSs) stained by BODIPY (magenta) and UV cones expressing GFP (green, *Tg(opn1sw1:GFP)*) in an 8 dpf larva. (F) Higher magnification sections from (E). Note that BODIPY stains the OSs of all photoreceptors, as well as the spot-like pocket of mitochondria immediately below the OS (Figures S1E–S1G). Note also that region-specific OS enlargements are restricted to UV cones. (G) Mean and 95% confidence intervals of UV cone OS lengths across the eye. V, ventral; SZ, strike zone; D, dorsal; N, nasal. Open-source 3D fish model created by M.Y. Zimmermann.

The ∼300-μm-diameter eyes of larval zebrafish necessarily offer limited spatial resolution (Haug et al., 2010), meaning that visually detecting their even smaller prey presents a substantial challenge. We therefore set out to determine the maximal number of UV cones the fish can use for this task. At 8 *dpf*, larval zebrafish have ∼2,400 UV cones per eye. These are unevenly distributed and exhibit a 3-fold elevation in the center of the SZ (Zimmermann et al., 2018), which in visual space is located at 38° azimuth and 27° elevation relative to the center of the monocular field (Figure S1A). At rest and during hunting, larval zebrafish converge their 169° ± 4.9° (n = 4) field-of-view eyes by ∼36° and ∼76° (Bianco et al., 2011, Patterson et al., 2013, Trivedi and Bollmann, 2013) to afford a frontal binocular overlap of 26° and 66°, respectively (Figures S1B–S1D). Based on these numbers, we computed the spatial detection limits of the UV detector array across both eyes (Figures 2A–2D and S1A–S1D).

Before initiating the actual strike, and prior to converging their eyes, zebrafish must first detect their prey (Gahtan et al., 2005, McElligott and O’malley, 2005). This mostly occurs within the upper visual field (∼30° elevation; Mearns et al., 2020), where the UV signal from paramecia is particularly prominent (Figures 1D and 1E). Within this region, prey-detection performance is highest when the target is laterally displaced from the center of the binocular visual field by ∼23° (Mearns et al., 2020). This same region was surveyed by each eye’s SZ (Figures 2A, S1A, and S1B), confirming that zebrafish indeed capitalize on the elevated UV cone density in this part of the eye for prey detection. However, with a mean SZ UV-cone spacing of 0.19 cones/°^2^ and a UV cone-receptive field diameter of ∼0.76°, even at its peak, this UV-detector array nevertheless dramatically undersamples visual space for this critical behavioral task (Figure 2B): larval zebrafish can detect <100-μm prey (Lawrence, 2007, Wilson, 2012) at up to 3.25 mm (Bianco et al., 2011) distance, where it subtends a visual angle of only 1.8°. This is more than two times smaller than required for reliable detection at the Nyquist limit. It therefore follows that zebrafish are unlikely to use more than a single UV cone at a time to trigger the initial behavioral response.

Once this prey is detected, zebrafish orient toward it and converge their eyes (Bianco et al., 2011, Gahtan et al., 2005, Jouary et al., 2016, McElligott and O’malley, 2005, Mearns et al., 2020, Patterson et al., 2013, Trivedi and Bollmann, 2013). This brings both SZs into near-perfect alignment directly in front of the fish, thus enabling stereoptic estimation of exact prey position for subsequent capture (Patterson et al., 2013; Figures 2D, S1C, and S1D). The actual strike is then initiated at a distance of ∼1 mm (Patterson et al., 2013), when a 100-μm paramecium subtends a visual angle of ∼5.7° (Figure 2D). At this angular size, it reliably covers two or three UV cones per eye yet rarely substantially more. Taken together, single UV cones in the SZ therefore likely underlie initial prey detection, triggering prey-orientation behavior. Further, the actual strike is then likely supported by at most a handful of UV photoreceptors per eye.

### UV Cone-Outer Segment Size Varies More Than 10-fold across the Eye

As single cones may suffice for prey detection, and in view of the relatively low UV signal in natural light (Losey et al., 1999, Zimmermann et al., 2018), UV cones in the SZ must be able to absorb photons with high efficiency to support hunting behavior. In contrast, UV cones outside the SZ might be able to afford lower photon catch probability and thus conserve space and energy, as it is possible to pool the coincident signals from multiple UV cones (e.g., for UV-dark silhouette-based predator detection) (Cronin and Bok, 2016). A simple way to increase a vertebrate photoreceptor’s photon catch probability is to enlarge its outer segment, which houses the phototransduction machinery (de Busserolles et al., 2014, Warrant and Nilsson, 1998). To test this, we genetically labeled all UV cones (green), stained outer segments of all cones using the membrane dye BODIPY (magenta), and assessed their morphology using confocal imaging (Figures 2E–2G). This revealed more than 10-fold variations in outer segment lengths. SZ UV cones had the longest outer segments (9.0 ± 0.4 μm), while the immediately neighboring ventral UV cones had the shortest (0.6 ± 0.8 μm). A secondary peak occurred in nasal UV cones (7.0 ± 0.5 μm), which survey the outward horizon, possibly to also support the UV-driven chromatic circuits in this part of the eye (Zimmermann et al., 2018). In cyprinid photoreceptors, photon catch probability (*F*) scales as a function of outer segment length (*l*) as

$$F=\frac{l\cdot k}{2.3+l\cdot k},$$

where *k* is the photoreceptor-type-specific absorption coefficient of 0.03^−μm^ (Warrant and Nilsson, 1998). Accordingly, the observed variation in outer segment length from 0.6 to 9.0 μm should lead to a ∼14-fold boost in photon catch probability for SZ cones. Together, the combination of UV cone density across the eye (factor 3), outer segment length (factor 14), and binocular superposition of the two eyes’ SZs during hunting (factor 2) should therefore lead to a 42- (monocular) to 84-fold (binocular with eyes converged) variation in UV sensitivity across the visual field.

Finally, located just beneath each outer segment, SZ UV cones also had consistently enlarged ellipsoid bodies (Figures 2F and S1E–S1G). These structures house the mitochondria that power phototransduction (Giarmarco et al., 2017, Okawa et al., 2008). Mitochondrial pockets might further act as micro-lenses to focus additional light onto outer segments (Knabe et al., 1997). With a more than 5-fold variation in ellipsoid body 2D area across the eye (Figure S1G), any such focusing effect would further boost UV-detection capacity of the SZ. We next asked how these anatomical differences might be reflected at the level of UV-light responses across the *in vivo* eye.

### SZ UV Cones Are Light Biased and Have a High Gain and Long Integration Times

**Figure 3:**
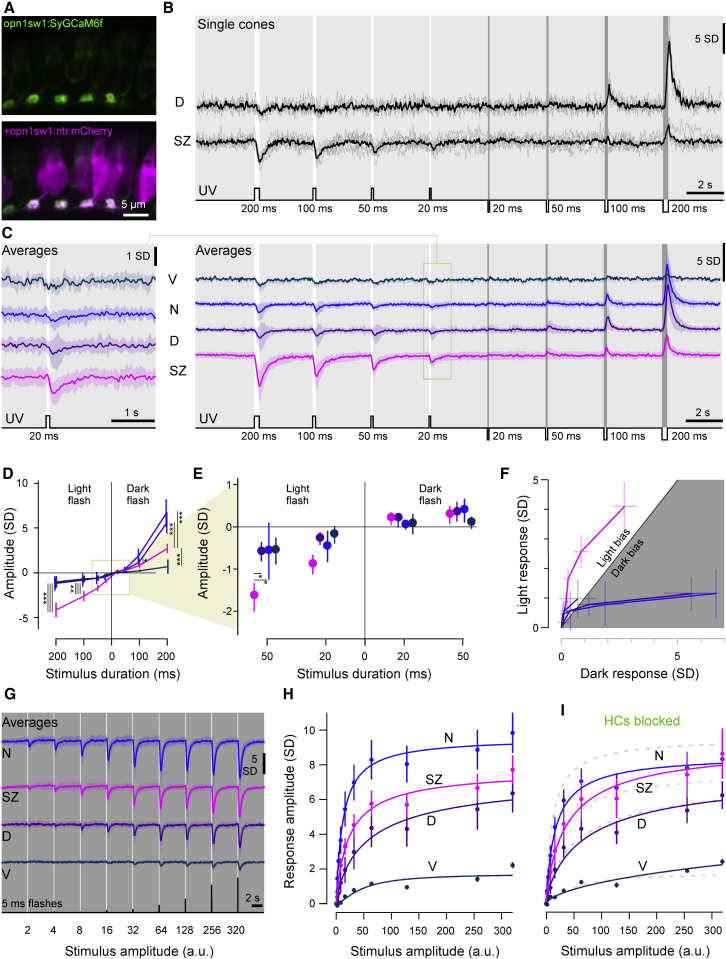
Imaging Cone Calcium in the Live Eye (A) Confocal images of synaptically targeted GCaMP6f (green, *Tg(opn1sw1:SyGCaMP6f)*) in UV cones (magenta, *Tg(opn1sw1:nfsBmCherry)*). (B) Mean and single trial dorsal and SZ single cone 2-photon calcium responses to varying duration light- (6 × 10^5^ photon/s/μm^2^) and dark-steps (0 photon/s/μm^2^) from a constant UV background (2.4 × 10^4^ photon/s/μm^2^). (C) Mean calcium responses to the same stimulus as in (B) from ventral, nasal, dorsal, and SZ cones (V, N, D, and SZ; n = 9, 21, 23, and 29, respectively). Shadings represent ±1 SD. Left panel shows an enlargement of the response to the 20-ms light step. (D) Mean and 95% confidence intervals of peak amplitudes from (C). (E) Enlargement from (D). All responses except nasal and ventral 20-ms dark-flash conditions were significantly different from zero (Mann-Whitney *U* test). Within-condition pairwise comparisons across for SZ versus the other three zones are indicated with asterisks (^∗^p < 0.05, ^∗∗^ p < 0.01, and ^∗∗∗^ p < 0.001, respectively; p value adjustment, Tukey method for comparing a family of four estimates). (F) Light and dark responses from (C) and (D) plotted against each other for equivalent stimulus durations, with 95% confidence intervals indicated. (G and H) Mean calcium responses to increasing-amplitude 5-ms light flashes from darkness, as indicated (G), and quantification (mean and 95% confidence intervals) with Hill functions fitted (H). (I) Quantification of calcium responses as in (G) and (H) following horizontal cell (HC) blockage using CNQX. For better comparison, curves from (H) are added as faint dashed lines. n = 51, 29, 46, and 17 for SZ, D, N, and V, respectively, for control and n = 51, 32, 46, and 19 after HC block.

To measure UV cone light responses *in vivo*, we expressed the synaptically tagged fluorescent calcium biosensor SyGCaMP6f (Dreosti et al., 2011) in all UV cone pedicles. We co-expressed mCherry (Shaner et al., 2004) under the same opn1sw1 promotor (Takechi et al., 2003) without synaptic tagging to reveal each cone’s full morphology and to confirm that SyGCaMP6f expression was restricted to the pedicles (Figure 3A). 7–8 dpf larvae were imaged under two photon at 64 × 16 pixel resolution (62.5 Hz), capturing one to five UV cone pedicles at a time. This allowed imaging light-driven cone-pedicle calcium in any part of the *in vivo* eye.

We first presented light and dark flashes from a constant UV background (STAR Methods). Prey-capture behavior can be initiated by the presentation of a bright spot as small as 2°, moving at a speed of 90°/s (Semmelhack et al., 2014). Such a moving stimulus activates a single UV cone for at most 30 ms if perfectly centered. At times, paramecia will however move somewhat slower (cf. Videos S1 and S2), meaning that also slightly longer stimulus durations are meaningful for prey detection. Accordingly, we presented light and dark flashes at varying durations. In an example recording, we observed that a SZ UV cone indeed responded to 20-ms and 50-ms UV-light flashes, while a dorsal cone failed to exhibit a detectable response (Figure 3B; Video S3). However, compared to the SZ UV cone, the dorsal UV cone responded much more strongly to a 200-ms dark flash.

Video S2 Example Prey-Capture Bout under UV, Related to Figure 1 Top-view of 7 *dpf* zebrafish larva mounted in agarose with eyes and tail free to move. Free-swimming paramecia appear as dark moving “dots”. Note prey-capture bout at t = 5 s.

Video S3 Imaging UV-Cone Synaptic Calcium *In Vivo*, Related to Figure 3 Calcium responses to bright- and dark-flashes in UV-cones from SZ (upper) and dorsal (D, bottom) as in Figure 3B. The video is an average of 5 repeats of single trial raw videos that were cropped and aligned. The magenta bar indicates the timing of bright and dark flashes.

Across multiple such recordings, SZ cones consistently responded strongly to light flashes (Figures 3C–3F), including to the 20-ms condition (Figures 3C and 3E), suggesting that SZ UV cones are indeed well suited to detect the presence of UV-bright prey. In contrast, dorsal and nasal UV cones were dark biased (Figures 3D and 3F), as would be useful to signal the presence of a UV-dark predator.

Next, we tested if variations in UV cone-outer segment lengths (cf. Figure 2G) could be linked to corresponding differences in the amplitudes of UV-light-evoked synaptic calcium signals. For this, we presented varying amplitude light flashes from darkness (Figure 3G). This confirmed that both SZ and nasal UV cones, which had the longest outer segments, also exhibited the largest synaptic calcium signals. To quantify these differences, we fitted a Hill function to each region’s stimulus-response curve (Figure 3H). We then determined the half-maximum response amplitude of ventral cones, which exhibited the smallest responses, and used this number to determine stimulus amplitudes that evoked equivalent-amplitude responses in the other UV cones (Figure 3H). Under this criterion, dorsal UV cones were ∼10-fold more responsive compared to ventral cones, followed by SZ UV cones (∼20-fold) and finally nasal UV cones (∼40 fold). Though qualitatively in line with anatomy, these effective gain changes at the level of synaptic calcium were generally larger than predicted and moreover did not directly scale with the relative distributions of outer segment lengths across the eye. This suggested that additional mechanisms may be at work. To test to what extent outer retinal inhibition may play a key role in defining the gain of UV cone synapses, we next pharmacologically blocked horizontal cells (HCs) using cyanquixaline (CNQX; STAR Methods). However, this circuit manipulation had no effect on the relative order of UV cone-response curves across zones and overall only resulted in minor amplitude variations (Figure 3J). Nevertheless, SZ and nasal UV cones now exhibited more similar response amplitudes, in line with their similar outer segment lengths. Remaining differences between the experimentally determined sensitivity of synaptic calcium responses (Figure 3J) and predictions from outer segment anatomy (Figure 2G) may be linked to a combination of non-linearities in the calcium biosensor (Chen et al., 2013, Dreosti et al., 2009), possible differences in synaptic calcium handling (Frank et al., 2009), and/or variations in phototransduction (see below).

**Figure 4:**
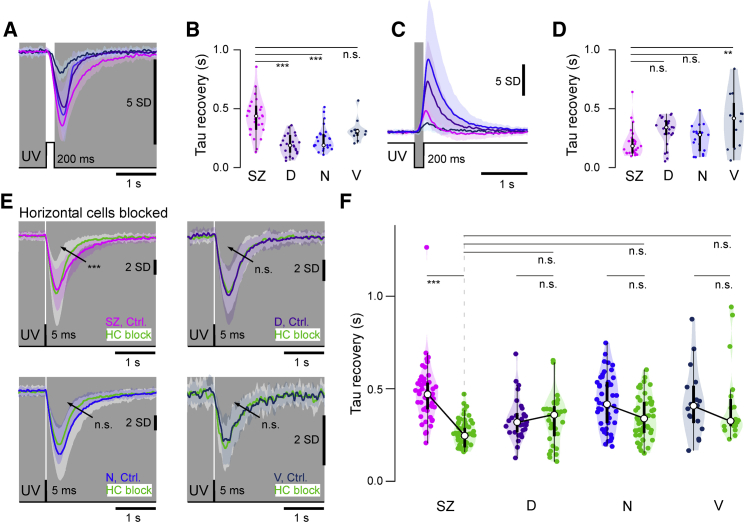
Temporal Tuning of UV Cones (A) Mean ± 1 SD responses to a 200-ms flash of light (6 × 10^5^ photon/s/μm^2^) from darkness (0 photon/s/μm^2^). (B) Box and violin plots of recovery time constants from (G). n = 29, 29, 23, and 13 for SZ, D, N, and V, respectively. (C and D) As in (A) and (B), but for an equivalent contrast dark flash. n = 27, 24, 19, and 13 for SZ, D, N, and V, respectively. ANOVA test ^∗^p < 0.02, ^∗∗∗^p < 0.0001 (H and J). n.s., not significant. (E) Mean ± 1 SD (shadings) calcium responses to a 5-ms light flash from darkness before (shades of purple) and after HC blockage using CNQX (green). (F) Quantification of recovery time constant after a 5-ms UV flash at 10^4^ photons/cone. n = 51, 29, 46, and 17 for SZ, D, N and V, respectively for the control condition and n = 51, 32, 46, and 19 after HC block. ANOVA test ^∗∗^p < 0.01, ^∗∗∗^p < 0.001. n.s., not significant.

Next, also calcium kinetics varied between UV cones. Specifically, SZ UV cones were particularly slow to recover back to baseline following a light flash (Figures 4A and 4B). This prolonged response might aid temporal signal integration across multiple SZ UV cones by postsynaptic circuits as the image of prey traverses the photoreceptor array. In contrast, recovery from dark-flash responses was either similar or even slightly faster compared to the rest of the eye (Figures 4C and 4D). To explore possible mechanisms underlying the slow recovery kinetics of SZ UV cones, we again blocked HCs. This revealed that unlike for UV cone amplitudes (cf. Figure 3J), UV cone kinetics were markedly affected by this manipulation (Chapot et al., 2017a) and in a region-specific manner (Figures 4E and 4F). Without feedback from HCs, the recovery kinetics of SZ UV cones was markedly sped up, while other UV cones were not significantly affected.

### UV-Dependent Prey Detection Is Difficult Outside the SZ

**Figure 5:**
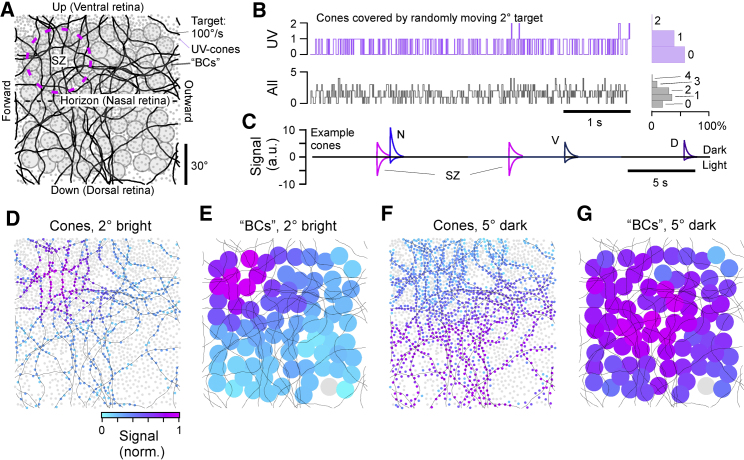
A Model of UV Cone Activation by a Small Moving Target (A) Model setup. Monocular UV cone distribution across the visual field (gray dots) with model bipolar cell (BC) array superimposed (filled circles) and target paths black line. The SZ was centered in the upper left quadrant, corresponding to the upper frontal visual field. (B) Number of cones touched by a moving 2° plotted as a trace over time, with histogram to the right. Top: UV cones. Bottom: any-type cone. (C) Time trace of model cone activation of four example cones, taken from representative regions across the array. Responses below and above zero correspond to activation in response to a 2° bright and 5° dark target, respectively. (D) Maximal activation levels of each cone over the full path for a 2° bright target, normalized to peak activation across the entire array. (E) Activation of BCs driven by UV cones in (D). (F and G) As in (D) and (E) but for a 5° dark target.

Combining our data from the UV cone distributions and *in vivo* response properties, we set up a simple linear model to estimate how different types of UV stimuli can be detected by the larval zebrafish’s monocular UV-detector array (STAR Methods). For this, we first recorded the position of every UV cone in a single eye and projected their 0.76° receptive fields into visual space (Figure 5A; cf. Figures 2A, 2C, S1A, and S1C). We next computed a series of random-walk stimulus paths across this array by an assumed bright 2° target moving at an average speed of 100°/s and with approximately naturalistic turning behavior (Jung et al., 2014, Shourav and Kim, 2017). This simulation confirmed our previous calculation that a single such target almost never (<0.1% of the time) covers two UV cones at a time (Figure 5B). In fact, most of the time (>60%), it covers zero UV cones, as it slips through gaps in the detector array. Even when adding all non-UV cones (STAR Methods), the maximal number of cones of any type covered at a time was three, with a single cone being the most likely incidence (∼40%; Figure 5B, bottom).

We then assigned response amplitudes and decay time constants for both light and dark flashes based on our calcium imaging results to each UV cone receptive field based on their position in the eye (Figure 5C, *cf.* Figure 3D; STAR Methods). For this, we also computed how an identically moving but larger (5°) dark target, meant to mimic a small or distant predator, activated UV cones. In the model responses shown, single example cones from different parts of the retina responded sparsely to either object as it traversed their receptive fields. The model clearly predicted that the light object would be most detectable in the SZ (Figure 5D; Video S4). Adding even small amounts of noise would rapidly make all but SZ-based UV-light detection of this nature almost impossible. Any detectability difference would be further enhanced by a population of postsynaptic bipolar cells (BCs), here modeled to simply sum the signals from all UV cones within a fixed radius. By integrating across more than one UV cone, BCs also capitalize on the slower light recovery times of SZ UV cones (Figure 5E; cf. Figures 4A and 4B; STAR Methods). In contrast, the large dark object moving along the same path was detectable across the entire array (Figure 5F). Here, the somewhat larger response amplitudes of dorsal UV cones were approximately compensated for by the relatively greater number of UV cones in the ventral half of the retina. This yielded an approximately homogeneous dark response at the level of BCs across the entire visual field (Figure 5G).

Video S4 A Model of Visual Detectability of Bright and Dark Moving Objects, Related to Figure 5 Left, modelled UV-cone detector array (top) and bipolar cells (bottom) responding to a bright 2˚ target moving in a pseudorandom path at 100˚/s. The target is meant to mimic a paramecium. Right, as left, with target size increased to 5˚ and contrast inverted to dark. The target is meant to mimic a distant or small predator. In each case, the colour-scaling indicates relative activation of cones or bipolar cells scaled to the array’s maximum. Note that the small light target is only readily detectable in the strike zone (top left in each array), while the predator is always detectable. Played back at real-time.

Taken together, the combination of differences in UV cone density (Figure 2A), outer segment size (Figures 2E–2G), and *in vivo* response properties at the level of presynaptic calcium driving release (Figures 3 and 4) therefore strongly suggests that detection of paramecia using the UV-detector array will be strongly and specifically facilitated in the SZ and perhaps all but impossible in most other parts of the visual field.

We next explored the mechanisms underlying the dramatic shift in response preference toward light stimuli by SZ UV cones. For this, we returned to *in vivo* recordings of light-driven calcium across the eye.

### Differences in Calcium Baseline Drive Differential Light-Dark Responses

**Figure 6:**
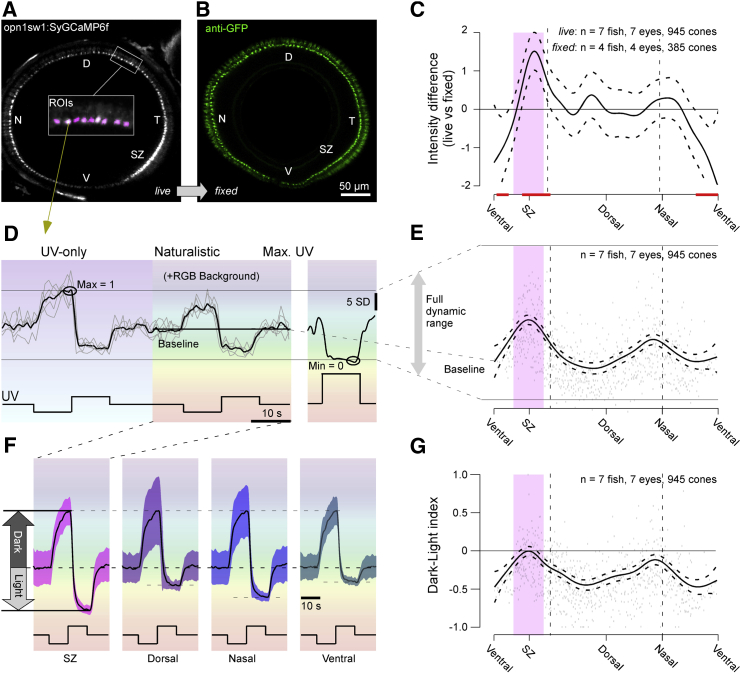
Calcium Baseline Predicts Dark-Light Responses (A and B) Whole-eye sagittal view of UV cone SyGCaMP6f in live *Tg(opn1sw1:SyGCaMP6f)* zebrafish under 3 × 10^5^ photon/s/μm^2^ UV background light (A) and after immunostaining against SyGCaMP6f using anti-GFP antibody (B). (C) Mean and 95% confidence intervals of the difference between live SyGCaMP6f signal per cone as in (A) and fixed signal as in (B), with red lines indicating regions that were significantly different from zero. (D) Example mean and individual trial single cone response to 0 photon/s/μm^2^ dark and 6 × 10^5^ photon/s/μm^2^ light steps from a constant brightness UV 3 × 10^5^ photon/s/μm^2^ without and with spectrally broad background light. After five repeats, a 1.5 × 10^7^ photon/s/μm^2^ UV light step was presented to drive calcium to a minimum (right). (E) Mean and 95% confidence interval of calcium baseline relative to the full dynamic range as indicated, with single datapoints in the back. (F) Mean ± 1 SD calcium responses to light and dark contrasts with naturalistic RGB background light across all UV cones in specified regions. Traces were shifted and scaled to align the baseline and peak dark response. (G) Mean and 95% confidence intervals of dark-light index (DLi) with single datapoints in the back.

To simultaneously record from all ∼120 UV cone pedicles in the sagittal plane at single-synapse resolution, we turned to higher-spatial-resolution scans of the full eye (STAR Methods). In this configuration, the basal brightness of the SyGCaMP6f signal under a constant UV background was consistently elevated in the SZ (Figure 6A). This brightness gradient was not related to differential SyGCaMP6f expression levels. When the same animal was fixed following live imaging and stained against the GFP fraction of SyGCaMP6f, the regional brightness differences were gone (Figure 6B). This suggests that the SyGCaMP6f signal elevations in the live eye were linked to constitutive variations in UV cone pedicle calcium baseline (Figure 6C). We therefore further explored how calcium baseline varies between UV cones and how this in turn might affect their ability to encode light and dark stimuli.

To explore this idea, we presented a simple step stimulus with UV light varying from 0% to 100% contrast around a mean background of 50% contrast (Figure 6D; Video S5). On every other repetition, this UV stimulus was superimposed on a naturalistic red-green-blue (RGB) background based on previous measurements of the spectrum of light in the zebrafish natural habitat (STAR Methods). Finally, at the end of n = 5 complete cycles, we presented a single, very bright UV-light flash to drive calcium to its light-evoked minimum. From here, we computed each UV cone’s full dynamic range as the SyGCaMP6f-signal difference between the periods when all lights are off (maximal calcium) and when all lights are on (minimal calcium). Relative to this full dynamic range, we then computed each cone’s baseline during naturalistic stimulation when UV light was held at 50% contrast. The resultant estimate of the calcium baseline across the eye recapitulated the previously observed brightness differences in the unstimulated eye. Signal baseline was maximal in the SZ, followed by a second, shallower peak around the nasal horizon (Figure 6E; cf. Figure 6C).

Video S5 Whole-Eye Imaging of Light-Driven UV-Cone Calcium Levels, Related to Figure 6 UV-cone calcium responses to bright- and dark-flashes as in Figure 6. The video is an average of 7 repeats of single trial raw videos that were cropped and aligned. The bars on the right indicate the timing of bright and dark flashes and the RGB background, which are all superimposed on a constant UV background (not indicated).

Next, we specifically compared response amplitudes to the 0% and 100% UV-contrast flashes during naturalistic background illumination in different zones. Like calcium baselines, this clearly showed that light and dark responses on average were most balanced in the SZ, followed by the nasal horizon, while both dorsal and ventral UV cones were strongly dark biased (Figure 6F).

To quantify this light-dark preference behavior, we calculated a dark-light index (DLi) from each cone (Figure 6G; see STAR Methods), where a *DLi* of −1 indicates that a cone exclusively responds to the dark step, while a DLi of 1 corresponds to a fully light-biased response. A DLi of 0 denotes equal responsiveness to dark and light steps. This revealed that DLi varied with eye position, with the most balanced responses observed in the SZ and near the nasal horizon, recapitulating the previously observed gradual variations in calcium baseline (Figure 6G; cf. Figures 6C and 6E) and response properties (cf. Figure 3F). In contrast, ventral and dorsal regions had a consistently negative DLi.

When compared directly, calcium baseline and DLi were strongly correlated (ρ = 0.85): a higher calcium baseline predicted a higher DLi (Figures S2A–S2D). UV cones from different eye regions simply occupied different ranges of what appeared to be one continuum linking DLi and baseline. Taken together, our whole-eye imaging data therefore strongly suggest that systematic variations in calcium baseline are closely linked to a UV cone’s preference for light or dark contrasts.

### HCs Do Not Underlie Regional Variations in DLi

Differences in calcium baseline across UV cones might be driven by differences in cone-intrinsic properties or differential interactions with HCs (Chapot et al., 2017b, Van Hook et al., 2019, Klaassen et al., 2012, Thoreson and Mangel, 2012). In the latter case, possible variations in the strength of a tonic inhibitory input from HCs (cf. Figures 3H, 3J, 4E, and 4F) might drive variations in cone baseline and thus DLi. If this were the case, then blockage of HC feedback should specifically elevate the low DLi of the dorsal and ventral retina. However, if anything, the opposite was observed. Pharmacological blockage of HCs did not elevate dorsal or ventral DLi, but instead slightly elevated DLi near the SZ and decreased it at the nasal horizon (Figures S2E and S2F; STAR Methods). Accordingly, unlike for response kinetics (cf. Figures 4E and 4F), it is unlikely that HCs strongly contribute to the observed shift in DLi among UV cones. Instead, intrinsic differences in the properties of each UV cone are likely dominant. What are these differences?

### Differential Expression of Phototransduction Cascade Genes Is Linked to Multiple Aspects of Regional UV Cone Tuning

**Figure 7:**
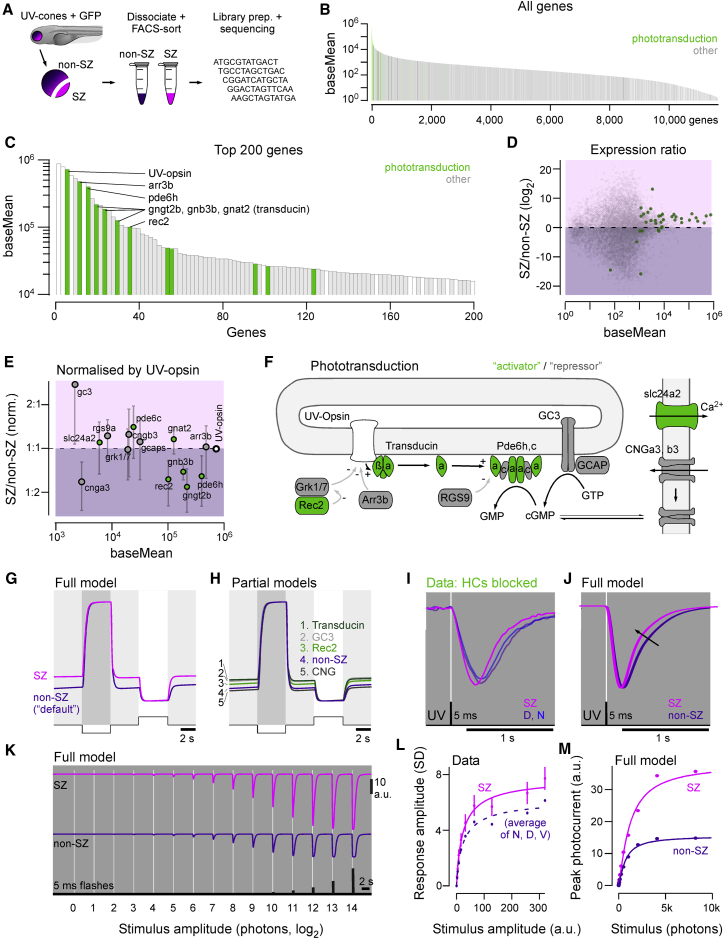
Tuning of Phototransduction Cascade Elevates SZ Baseline (A) UV cone RNA sequencing (RNA-seq) workflow. Retinas from 7 dpf zebrafish *Tg(opn1sw1:GFP)* were dissected and separated into SZ and non-SZ. After cell dissociation, UV cones were FACS sorted and immediately flash frozen. Samples were then subjected to library preparation for next-generation sequencing. (B and C) All detected genes in UV cones ranked by expression label, with phototransduction genes highlighted (B), and zoom in to the top 200 genes (C). The two most highly expressed genes are both non-protein-coding genes; therefore, UV opsin is the highest expressed protein-coding gene. (D) Mean gene expression ratio between SZ and non-SZ batches, with phototransduction genes highlighted. (E) As in (D), but normalized to UV-opsin expression level in each batch and zoomed in to high expression phototransduction targets. Green and gray markers denote activators and repressors of the photo-response, respectively. Error bars represent SEM. (F) Schematic of phototransduction based on Yau and Hardie (2009), with activators and repressors denoted in green and gray, respectively. (G) Simulated current response of SZ and non-SZ UV cones to 100% dark and light contrasts from a 50% contrast background based on Invergo et al. (2014). Non-SZ was based on default model parameters, while SZ uses relatively scaled parameters according to gene expression ratios as in (E). (H) Effects of expression changes of individual phototransduction components compared to non-SZ. (I) Mean calcium responses to a flash of light from darkness in SZ, nasal, and dorsal UV cones from Figure 4E. (J) Output of full phototransduction model to an equivalent stimulus between SZ and non-SZ batches. (K) Full model output to a series of increasing amplitude 5-ms light flashes from darkness for SZ and non-SZ batches. (L and M) Stimulus-response data from SZ and average of non-SZ data (N+D+V) from Figure 3H (L) and corresponding quantification of the phototransduction model output (K) (M).

To pinpoint intrinsic differences between UV cones that might underlie the observed regional differences among UV cone functions, we used a transcriptomics approach (Stark et al., 2019). For this, we dissected entire retinas expressing GFP in all UV cones and surgically separated the SZ from the remainder of the retina (non-SZ). We then dissociated and FACS-sorted UV cones for subsequent transcriptomic profiling (Figure 7A; STAR Methods). Genes involved in phototransduction dominated the transcriptome of both SZ and non-SZ batches, with UV-opsin being the most strongly expressed protein-coding gene (Figures 7B and 7C). Phototransduction genes were generally more highly expressed in SZ batches (Figure 7D), consistent with their larger outer segment sizes (cf. Figure 2). Accordingly, to compare the relative expression of key phototransduction genes, we normalized the expression level of each gene by the respective UV opsin expression level in each sample (Figure 7E). This revealed that some key phototransduction genes had relatively higher expression in the SZ (e.g., gc3), while others were downregulated (e.g., cnga3 or gngt2b). Building on our exquisite understanding on phototransduction in general (Fain et al., 2010, Lamb, 2013, Pergner and Kozmik, 2017, Pugh and Lamb, 1993, Yau and Hardie, 2009), each of these regulatory changes can be linked to a specific functional effect (Invergo et al., 2013, Invergo et al., 2014).

To quantitatively explore how the sum of all relative gene expression changes might affect the interplay of activators and repressors of the phototransduction cascade (Hurley, 1987, Pugh and Lamb, 1993, Pugh et al., 1999), we used a computational model of phototransduction in ciliary photoreceptors (Invergo et al., 2013, Invergo et al., 2014; Figure 7F). We kept all preset parameters of the model constant and only adjusted the relative levels of phototransduction elements according to the observed expression differences between SZ and non-SZ batches. In this way, we tested if we could turn a non-SZ cone (default model) into a SZ cone through specific regulatory manipulations. Indeed, altering only the top four most differentially expressed targets (transducin, GC3, recoverin 2 [Rec2], and CNG) phenomenologically reproduced the elevation in constitutive baseline and corresponding increase in the amplitude of the light response in SZ cones (Figure 7G). The modulation of single gene products’ relative expression levels could have major effects, most notably in the case of transducin and GC3 and to a lesser extent also for Rec2 (Dizhoor et al., 1991, Zang et al., 2015; Figure 7H). Interestingly, transducin expression is also systematically adjusted between peripheral and foveal L/M cones in the primate (Peng et al., 2019), indicating that this might constitute a regulatory hotspot for tuning cone function.

We next used the same phototransduction model to also explore any possible effects of molecular tuning on the kinetics (Figures 7I and 7J) and gains (Figures 7K–7M) of UV cone responses. Here, our phototransduction model predicted slightly faster recovery kinetics of SZ compared to non-SZ batches (Figure 7J; cf. Figure 7I). Finally, our phototransduction model also predicted a ∼3-fold gain change in SZ UV cones compared to the remainder of the eye (Figures 7K and 7M), which qualitatively captured a small gain increase when comparing calcium-imaging data from SZ UV cones to the mean of gains across nasal, ventral, and dorsal UV cones (Figure 7L). This tentatively suggests that phototransduction tuning might be one additional factor that ultimately leads to the substantial UV cone gain differences observed across at the level of presynaptic calcium (Figures 3G and 3H).

Taken together, our transcriptomics data and phototransduction model therefore strongly suggest that diverse aspects of the eye-wide functional heterogeneity among UV cones (cf. Figures 3, 4, and 6) can be linked to differential regulation of phototransduction (Figure 7).

### Imaging Synaptic Release from Cones *In Vivo*

We next asked if and how the observed variations in UV cone synaptic calcium are translated into rates of light-driven synaptic vesicle release in the live eye.

**Figure 8:**
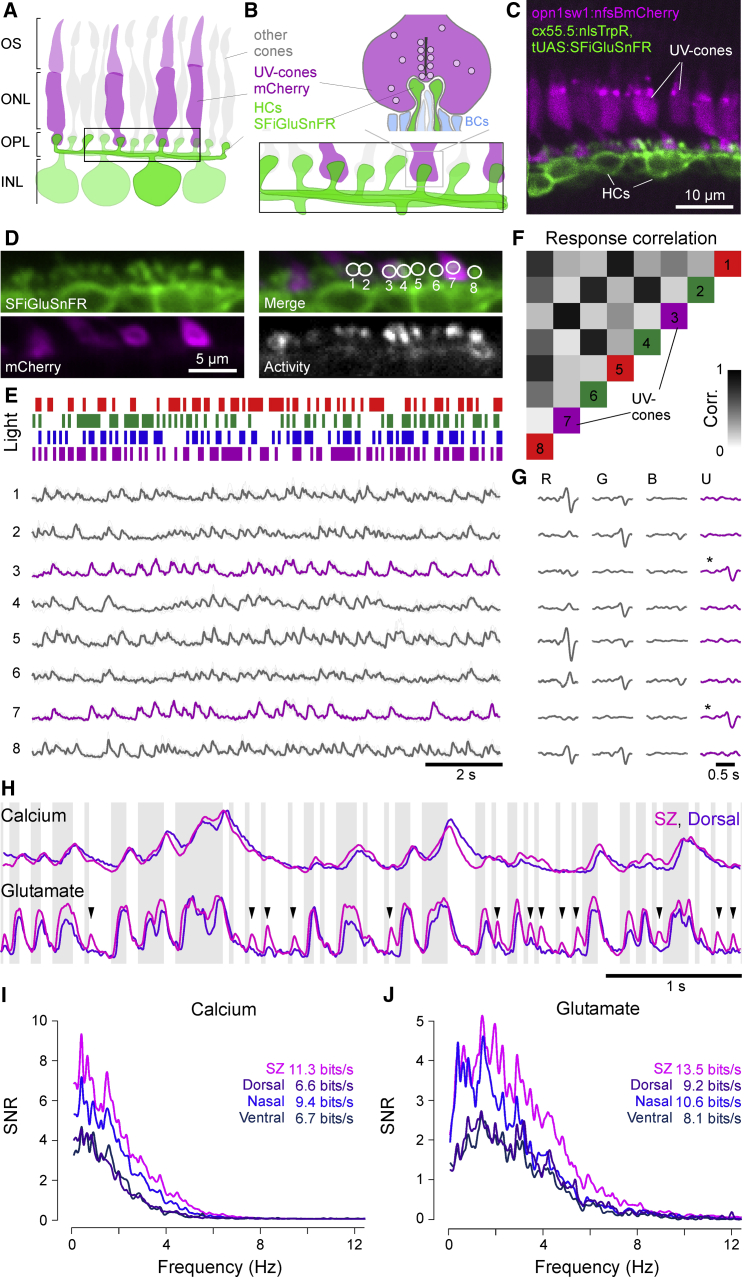
Synaptic Release Accentuates Functional Differences between UV Cones (A and B) Schematic of HC dendrites at photoreceptor synaptic invaginations. SFiGluSnFR expression in HC dendrites is well positioned to detect glutamate release from ribbon synapses (bar structure) at single terminals of any cone type. UV cones are identified by co-expression of mCherry as before. OS, outer segment; ONL, outer nuclear layer; OPL, outer plexiform layer; INL, inner nuclear layer. (C) *In vivo* two-photon image of SFiGluSnFR in HCs and nfsBmCherry in UV cones. (D) Scan field for SFiGluSnFR recordings. Individual HC dendritic bundles at single cone terminals are readily visible. ROIs 3 and 7 are associated with UV cones as seen by overlap with the mCherry signal. A map of pixel-to-pixel correlation over time (Franke et al., 2017) highlights localized activity at each cone terminal. (E) Partial example trace of mean and individual trial glutamate responses of ROIs from (D) to a tetrachromatic binary noise stimulus (STAR Methods). UV cone responses highlighted in magenta. (F) Correlation of glutamate responses across pairs of ROIs. ROIs 3 and 7 are highly correlated only to each other. Color code is based on each ROI’s preferred response as in (G). (G) Linear filters (“kernels”) recovered by reverse correlation of each ROI’s response to the noise tetrachromatic stimulus (E). R, G, B, and U denote red, green, blue, and UV light, respectively. UV cones are highlighted by asterisks. (H) Partial example trace of mean calcium (SyGCaMP6f) and glutamate (SFiGluSnFR) responses of SZ and dorsal UV cones to the tetrachromatic noise stimulus. Background shading indicates UV light and dark stimulus periods. Arrowheads highlight enhanced glutamate response transients from SZ relative to dorsal UV cones. (I) Signal-to-noise ratio in the Fourier domain and resulting information rate in calcium responses across UV cones from different regions. (J) As in (I), computed for glutamate responses. n = 35, 20, 28, and 18 for calcium in SZ, D, N, and V, respectively, and 51, 20, 22, and 18 for glutamate SZ, D, N, and V, respectively.

To address this question, we established optical glutamate recordings from single cones in the live eye by expressing the fluorescent glutamate biosensor SFiGluSnFR (Marvin et al., 2018) in postsynaptic HCs. HCs contact cones at specialized invaginations that provide a partial diffusion barrier against the extracellular matrix (Chapot et al., 2017a, Regus-Leidig and Brandstätter, 2012), meaning that their dendrites can act as specific and spatially restricted glutamate antennas (Chapot et al., 2017a). As a population, HCs contact all four types of cones in the zebrafish eye (Klaassen et al., 2016, Li et al., 2009, Yoshimatsu et al., 2014), meaning that only a subset of HC dendritic signals correspond to synaptic release from UV cones. To identify these contacts, we co-expressed mCherry in UV cones (Figures 8A–8C). In an example recording from the nasal retina, we presented a 12.8-Hz tetrachromatic binary noise stimulus (Zimmermann et al., 2018; STAR Methods) and recorded the glutamate signals from the HC dendrites that innervate a row of neighboring cones (Figure 8D; Video S6). Among eight example regions of interest (ROIs), each covering a presumed single cone’s output site, two were identified as UV cones based on mCherry co-expression (ROIs 3 and 7). Across glutamate responses within all eight ROIs (Figure 8E), example sections of traces extracted for the two UV cones were very similar to each other but distinct from all other traces (Figures 8E and 8F). Moreover, reverse correlation of each ROI’s response to the noise stimulus revealed a pronounced UV component for the two UV cones but diverse non-UV components in all other sites (Figure 8G). This strongly indicated that there was no glutamate spillover between neighboring ROIs (see also discussions in Chapot et al., 2017a, Franke et al., 2017, James et al., 2019). Our approach therefore allowed recording UV cone-driven glutamate in the live eye at single-pedicle resolution. We next used this approach to compare UV cone calcium-to-glutamate transfer functions in different parts of the eye.

Video S6 Imaging Glutamate Release from Cones *In Vivo*, Related to Figure 8 Video of mean glutamate responses over n = 7 repetitions of the tetrachromatic binary noise stimulus as in Figure 8. Green is SFiGluSnFR in HC and red is mCherry expression in UV-cones. The bars on the right indicate the timing of flashes of each LED.

### Glutamate Release Accentuates Existing Differences in Presynaptic Calcium

In nature, photoreceptors are constantly exposed to a rapidly changing stream of light- and dark-events as animals explore their visual environment. To explore how UV cones in different parts of the eye differentially encode complex light-dark sequences, we recorded calcium and glutamate responses to the tetrachromatic binary noise-stimulus. Superimposition of the average calcium (top) and glutamate (bottom) responses to this stimulus from SZ and dorsal UV cones revealed a marked difference in the synaptic transfer between these zones (Figure 8H; Video S7): Despite relatively similar responses at the level of calcium (top), only SZ UV cones responded strongly to the most rapid of stimulus reversals (bottom, arrowheads). These differences, which could not be explained by differences in the kinetics of GCaMP6f (Chen et al., 2013) and SFiGluSnFR (Armbruster et al., 2019, Marvin et al., 2018; Figure S3A), were subtly visible at the level of calcium, but they were strongly accentuated at the level of glutamate. Qualitatively similar effects were observed across all four zones (Figures S3B–S3E).

Video S7 Glutamate Release Differences between SZ and Dorsal, Related to Figure 8 Video of mean glutamate responses over n = 4 repetitions of the tetrachromatic binary noise stimulus as in Figure 8. Green is SFiGluSnFR in HC and red is mCherry expression in UV-cones. Circles indicate UV-cone terminals shown in the bottom as high-magnification. The bars on the right indicate the timing of flashes of each LED.

To determine how these differences can be linked to the amount of information which can be linearly decoded from the responses in each case, we computed the information rate based on the signal-to-noise ratio (SNR) in the Fourier domain (Figures 8I and 8J; van Hateren and van der Schaaf, 1998). Both at the level of calcium and glutamate, the most linearly decodable information was found in SZ cones, followed by nasal, dorsal, and finally ventral UV cones. Moreover, the more pronounced glutamate responses of the SZ to rapid stimulus changes led to a higher SNR, especially in the higher frequency domain. Accordingly, our glutamate imaging experiments suggest that the differential tuning of UV cones at the level of anatomy (Figure 2), phototransduction (Figure 7), inputs from surrounding inhibitory networks (Figures 3 and 4), and synaptic calcium (Figures 3, 4, 5, and 6) is preserved and possibly even enhanced at the level of synaptic release (Figure 8).

## Discussion

We have shown that larval zebrafish may use single UV cones at a time to detect the UV-bright microorganisms they feed on (Figures 1 and 5). For this, UV cones in the retina’s SZ are particularly dense and exhibit grossly enlarged outer segments (Figures 2 and S1) to boost local UV-photon detection probability. This is complemented by an elevation in these UV cones’ synaptic calcium baseline (Figures 3 and 6) that likely stems from molecular retuning of the phototransduction machinery (Figure 7). In addition, HCs selectively slow down SZ UV cone recovery kinetics following a flash of light (Figure 4). Together, this leads to an increased dynamic range for encoding UV-bright events (Figure 3) and sets of the capacity for increased information transfer across the synapse at the level of vesicle release driving retinal circuits (Figure 8). UV cones in the SZ are therefore exquisitely tuned to support the visual detection of prey. In contrast, the remainder of the UV-detector array is less dense and uses smaller outer segments and a lower calcium baseline to detect large UV-dark objects, such as predators. In doing so, non-SZ UV cones signal more sparsely and presumably conserve energy.

### Mechanisms of Photoreceptor Tuning in Vertebrates

For all we know, all sighted vertebrates have at least a mild form of an area temporalis or area centralis, and in some species, such as many primates as well as birds of prey and species of reptiles and fish, these specialized regions have further evolved into a fovea (Bringmann, 2019, Bringmann et al., 2018, Collin et al., 2000, Land, 2015). However, data on the possibility of regional tuning of photoreceptor function across most of these species remain outstanding with the notable exception of primates (Baudin et al., 2019, Sinha et al., 2017), mice (Baden et al., 2013b), and now zebrafish. In each of these latter three, cone function has been found to be regionally tuned.

In many ways, both the “purpose” of functional tuning of SZ UV cones and the underlying cellular and molecular mechanisms are reminiscent of differences between peripheral and foveal cones of the primate retina (Baudin et al., 2019, Curcio et al., 1990, Kemp et al., 1988, Mowat et al., 2008, Peng et al., 2019, Sinha et al., 2017). For example, in both zebrafish SZ UV cones and primate foveal cones, outer segments are elongated (Curcio et al., 1990, Packer et al., 1989) and light-response kinetics are slowed (Baudin et al., 2019, Sinha et al., 2017). In the primate fovea, expression of rod-transducin gamma subunit has been discussed as one determinant of the slowed kinetics (Peng et al., 2019), which conceptually links with our finding of reduced levels of cone-transducin gamma subunit in zebrafish SZ cones. In each case, these structural and functional alterations can be linked to an increased capacity for the detection of low numbers of photons and subsequent signal processing. In the primate fovea, they are critical to keep noise at bay to supply a low-convergence postsynaptic retinal network (Ala-Laurila et al., 2011, Angueyra and Rieke, 2013). Establishing to what extent the postsynaptic networks in the zebrafish’s SZ resemble those of the primate fovea will be an important area of research in the future. Nevertheless, already now it seems clear that noise reduction will be an asset also for SZ UV cones. In contrast to primates and zebrafish, mice have only a very mild area centralis aligned with visual space above the nose (Bleckert et al., 2014, Dräger and Olsen, 1981, Salinas-Navarro et al., 2009). However, they feature a pronounced opsin expression gradient across the retina’s dorsal-ventral axis (Szél et al., 2000), which has been linked to differential processing of light and dark contrasts (Baden et al., 2013b), much in line with observed differences in zebrafish UV cones. However, unlike in zebrafish, ventral short-wavelength vision in mice is dark biased (Baden et al., 2013b), which rather hints at the flexibility in how photoreceptors can be tuned to support specific visual tasks.

For the most part, the detailed cellular and molecular mechanisms that lead to differential cone-tuning across the retinal surface in mice and primates remain to be established. Building on our work, we anticipate that the possibility to perform high-throughput *in vivo* experiments in genetically modified larval zebrafish will be a major asset for studying mechanisms of photoreceptor tuning in general.

### Asymmetric Outer Retinal Circuits Contribute to UV Cone Tuning

In the intact eye, photoreceptors rarely signal in isolation. Instead, in both invertebrates and vertebrates, they tend to be intricately interconnected with neighboring photoreceptors and/or local feedback circuits (Heath et al., 2020, Masland, 2001, Schnaitmann et al., 2018). Locally adjusting how surrounding circuits interact with individual photoreceptors therefore presents another potential mechanism for regional tuning. Here, we have shown in larval zebrafish, HCs differentially interact with UV cones in different parts of the eye (Figures 3G–3J, 4E, 4F, S2E, and S2F). In general, blocking HC circuits had little or no effect on UV cone functions in the dorsal and ventral retina (and generally only weak effects in the nasal retina), while in SZ UV cones, both recovery kinetics and response amplitudes were markedly modulated by HCs. In line, previous imaging work on mouse cones reported a general speeding up of cones in the absence of HCs. However, conversely, electrophysiological recordings from goldfish (Kamermans et al., 2001) and primate (Sinha et al., 2017) cones reported slowed responses in the absence of HC feedback. Notably, beside HCs, the zebrafish outer retina is also innervated by interplexiform cells (Esposti et al., 2013, Robles et al., 2014), which may play an additional role in shaping UV cone functions. To what extent regional effects of outer retinal decoupling in zebrafish generalize across other cone types or the dendrites of BCs, and if they can be linked to a putative difference in the functional distribution of HC circuits, will be important to assess in the future.

Next, if and how other cone photoreceptors may regionally interact with UV cones remains an open question. However, it seems unlikely that interactions with rod photoreceptors contribute strongly to UV cone tuning. First, at 7 dpf, zebrafish rod photoreceptors remain restricted to the dorsal and ventral poles of the eye (Zimmermann et al., 2018), precisely opposite to the distribution of HC influences on UV cones. Second, at this age, rods are thought to be immature (Branchek and Bremiller, 1984). Third, across vertebrates, including in adult zebrafish, rod functions tend to be more closely interlinked with the circuits and functions of red and green cones (Baden and Osorio, 2019, Behrens et al., 2016, Li et al., 2012).

### Synaptic Tuning through the Ribbon

Beyond altering the morphological and biochemical properties of the outer segment, our results further suggest that the pedicle is functionally adjusted to support distinct modes of calcium-dependent vesicle release in UV cones in different parts of the eye. Cones use ribbon-type synapses, which have been a key focus for investigating the functional tuning of neural circuits (Baden et al., 2013a, Bellono et al., 2018, Heidelberger et al., 2005, Lagnado and Schmitz, 2015, Moser et al., 2020, Regus-Leidig and Brandstätter, 2012, Sterling and Matthews, 2005, Thoreson, 2007, Wichmann and Moser, 2015). For example, electrosensory ribbon synapses in rays and sharks are differentially tuned at the level of both synaptic ion channels and ribbon morphology to support the encoding distinct signal frequency bands required by these two groups of animals (Bellono et al., 2018). Indeed, ribbon synapses across species and modalities support a vast range of functional properties, and generally, the structure and function of each group of synapses can be closely linked to specific signaling requirements (Heidelberger et al., 2005, Lagnado and Schmitz, 2015, Moser et al., 2020, Sterling and Matthews, 2005, Thoreson, 2007). While therefore ribbon synapses do strongly vary across distinct sets of neurons that support diverse functional tasks, to our knowledge, this type of tuning has not been studied across a single neuron type. Accordingly, in the future it will be important to establish if and how the observed differences in synaptic transfer functions across zebrafish UV cones can be linked to structural and molecular differences in the synapse itself.

### How to Detect Small and Moving UV-Bright Prey

As discussed, the image of UV-bright prey falling onto the back of the retina when zebrafish first detect it barely covers the size of a single UV cone’s outer segment. Yet, behaviorally, zebrafish only respond if the prey moves (Semmelhack et al., 2014), which ought to require at least two receptors to decode the angled space-time trajectory in the stimulus. This raises two key questions. How can the bright signal from a single UV cone be reliably propagated to postsynaptic circuits, and how can its motion be encoded?

Vertebrate photoreceptors are “off” cells, meaning that transmitting brief on events through their positively rectifying synapse can be challenging (Baden et al., 2013a, Heidelberger et al., 2005). Instead, cone ribbon synapses are particularly good at signaling sudden off events by way of a transient burst of near-instantaneous vesicle release following a period of refilling during the preceding on event (Thoreson, 2007). Perhaps counterintuitively, rather than attenuating this ribbon rebound, the slowed calcium light-recovery kinetics in SZ UV cones (Figures 4A–4D) might in fact serve to help to reduce calcium levels during the on event so as to facilitate refilling of the ribbon in this time. Once the prey target moves out of the UV cone’s receptive field, calcium can then rush back in and trigger a transient burst of vesicle release as an off event. In support, our glutamate imaging experiments (Figures 8 and S3) show that UV SZ cones are particularly good at encoding the transition from on to off events (e.g., Figure 8H). From here, it is tempting to speculate that the same mechanism might also serve to detect a nondirectional form of motion that does not categorically require a second photoreceptor. Instead, perhaps it is enough for the target to simply enter a UV cone’s receptive field and then to leave again, thus generating a transient and positive glutamate signal for postsynaptic circuits to process. Such a process could potentially even explain the velocity dependence of prey-capture behavior (Semmelhack et al., 2014); a too-fast target may not suffice to cause a sufficient drop in calcium, while a too-slow target would likely hamper the transience of the off signal, as release from on suppression may be too gradual. Similarly, it might go partway to explaining why prey-capture behavior can be elicited by darker than background targets (Bianco et al., 2011). In the future, it will be important to explore if and how this “rebound trick” is meaningfully used to drive inner retinal prey detection and circuits. For example, flashing a suitably sized and positioned stationary UV-bright spot on and off should in this case suffice to trigger prey-orientation behavior.

### Studying Prey-Capture Behavior in the Lab

Larval zebrafish prey-capture behavior has been extensively studied in the lab (Bianco et al., 2011, Gahtan et al., 2005, Jouary et al., 2016, McElligott and O’malley, 2005, Muto and Kawakami, 2013, Novales Flamarique, 2016, Patterson et al., 2013, Semmelhack et al., 2014, Trivedi and Bollmann, 2013), though never specifically under UV. Here, our behavioral experiments (Figure 1H) strongly suggest that it is the UV cones, rather than UV light per se, that provide the dominant inputs to larval zebrafish prey-capture circuits; even though the spectrum of our UV LED overlapped with the alpha band of blue opsin as well as the beta bands of red and green opsins, UV illumination in the absence of UV cones did not rescue the behavioral phenotype. This also rules out a major contribution of a possible chromophore shift from A1 to A2 in any-type cones (Enright et al., 2015, Suliman and Novales Flamarique, 2014), since unlike for long-wavelength opsins, UV-opsin action spectra for A1 and A2 are virtually identical.

Nevertheless, even under low-UV conditions or in the absence of UV cones, zebrafish continue to display some prey-capture behavior. This suggests that non-UV cones can, if required, feed into prey-capture circuits, perhaps to boost signal power in the absence of systematic background clutter, as is also the case under typical lab conditions. In support, the strong UV dominance in SZ BCs and RGCs is complemented by smaller signals elicited also at other wavelengths (Zhou et al., 2020, Zimmermann et al., 2018). In parallel, it is important to consider the specific absorption spectrum of the zebrafish UV opsin relative to the spectrum of any illuminating light. For safety reasons, commercially available thin-film transistor (TFT) monitors (Franke et al., 2019), projectors, and organic LED (OLED) screens used in behavioral experiments tend to restrict short wavelengths to <1% signal power below 420 nm. In contrast, zebrafish UV-opsin absorption peaks at 365 nm (Chinen et al., 2003, Robinson et al., 1993), meaning that the short-wavelength signal of most of these light sources will activate the UV opsin with <1% efficiency. Nevertheless, owing to the extremely high photon-catch efficiency of SZ UV cones, this might still generate a useful signal, provided the screen is sufficiently bright. Moreover, different projection setups (Bianco et al., 2011, Jouary et al., 2016, Semmelhack et al., 2014, Trivedi and Bollmann, 2013) or live paramecia illuminated by indoor lighting (Bianco et al., 2011, Gahtan et al., 2005, McElligott and O’malley, 2005, Patterson et al., 2013) or indeed a fluorescence microscope’s excitation light (Muto et al., 2013) might afford higher spectral overlap. In the future, it will therefore be critical to establish in more detail how the addition of UV light affects behavioral performance.

### Retinal and Central Wiring for Prey Capture

The region-specific differences in UV cone function present the first pre-processing steps to detect prey and predators already at the visual system’s first synapse (Baden et al., 2013b, Chapot et al., 2017a). However, how these signals are used by retinal and brain networks for robust extraction of such behaviorally crucial information remains an open question. Ultimately, light patterns picked up by distinct regions of the UV detector array must lead to the differential activation of brain circuits that control distinct behavioral programs (Dunn et al., 2016, Preuss et al., 2014, Semmelhack et al., 2014). For this, the signal must first travel to the feature extracting circuits of the inner retina (Baden et al., 2019, Masland, 2001) via the diverse set of retinal BCs (Connaughton and Maguire, 1998, Connaughton and Nelson, 2000, Li et al., 2012, Zimmermann et al., 2018). Previous work highlighted a strong dominance of inner retinal UV-on circuits specifically in the SZ (Zimmermann et al., 2018), suggesting that the signal from SZ UV cones is indeed selectively picked up by a subset of local UV-on BCs for further processing. Next, the UV signal must be selectively sent to the specific relevant processing centers of the brain (Connaughton and Nelson, 2015, Robles et al., 2014, Xiao and Baier, 2007). Here, recent work showed that also RGCs, whose axons form the optic nerve, are regionally tuned for prey capture in the SZ (Zhou et al., 2020). Like BCs, the vast majority of RGCs in the SZ are UV-on circuits, in line with the boost of lighter-than-background signals in SZ UV cones (Figures 3 and 6). Moreover, their UV-signals are markedly slowed compared to the remainder of the eye, tentatively suggesting that also kinetic differences first set up in UV cones (Figure 4) reliably propagate through the retinal network. Finally, pretectal arborization field 7 (AF7), which underpins prey-capture behavior, is mainly innervated by temporal, but not nasal, RGCs (Robles et al., 2014, Semmelhack et al., 2014), strongly hinting that AF7 may be predominately driven by SZ circuits. Clearly, circuits for prey capture in larval zebrafish are both anatomically (Robles et al., 2014, Semmelhack et al., 2014, Zhou et al., 2020) and functionally (Zhou et al., 2020, Zimmermann et al., 2018) regionalized to drive a regionally biased behavioral repertoire (Bianco et al., 2011, Mearns et al., 2020). To what extent this can be supported through regional tuning of neuron types alone, as in case of UV cones, or in addition requires the specific positioning of unique neuron types in different parts of the eye and brain will be important to address in the future. Indeed, transcriptomic analysis recently highlighted the putative presence of one “extra” BC type specifically in the primate fovea (Peng et al., 2019), with yet-unknown morphology and function.

## STAR★Methods

### Key Resources Table

**Table:** 

REAGENT or RESOURCE	SOURCE	IDENTIFIER
**Antibodies**		

Chicken anti-GFP	AbCam	Cat#13970; RRID:AB_300798
Rabbit anti-cox iv	AbCam	Cat#Ab209727
Donkey anti-rabbit Alexa647 dye conjugate	ThermoFisher	Cat#A-21244
Donkey anti-chicken IgG CF488A conjugate	Sigma	Cat#SAB4600031; RRID:AB_2721061

**Chemicals**		

Paraformaldehyde	Agar Scientific	Cat#R1026
Triton X-100	Sigma	Cat#X100
Hoechst 33342	Invitrogen	Cat#H21492
BODIPY	Invitrogen	Cat#C34556
1-phenyl-2-thiourea	Sigma	Cat#P7629
α-bungarotoxin	Tocris	Cat#2133
Agarose low melting	FisherScientific	Cat#BP1360-100
CNQX	Tocris	Cat#1045

**Deposited Data**		

2-photon imaging data	This manuscript	https://datadryad.org/stash/landing/show?id=doi%3A10.5061%2Fdryad.w0vt4b8n3 https://www.badenlab.org/resources http://retinal-functomics.net/
Cone-density counts	This manuscript	https://datadryad.org/stash/share/IIkIQPdIivCdPqjTc44A-aqCLTWQjBADqV4zZmWDCi8 https://www.badenlab.org/resources http://retinal-functomics.net/
Natural imaging data	This manuscript	https://datadryad.org/stash/landing/show?id=doi%3A10.5061%2Fdryad.w0vt4b8n3 https://www.badenlab.org/resources http://retinal-functomics.net/
Transcriptome data	This manuscript	https://datadryad.org/stash/landing/show?id=doi%3A10.5061%2Fdryad.w0vt4b8n3 https://www.badenlab.org/resources http://retinal-functomics.net/
Associated summary statistics	This manuscript	https://datadryad.org/stash/landing/show?id=doi%3A10.5061%2Fdryad.w0vt4b8n3 https://www.badenlab.org/resources http://retinal-functomics.net/

**Experimental Models: Organisms/Strains**		

Danio rerio (zebrafish): *Tg(opn1sw1:nfsBmCherry)*	Yoshimatsu et al. (2016)	N/A
Danio rerio (zebrafish): *Tg(opn1sw1:GFP)*	Takechi et al. (2003)	N/A
Danio rerio (zebrafish): *Tg(opn1sw1:GFP:SyGCaMP6f)*	This manuscript	N/A
Danio rerio (zebrafish): *Tg(cx55.5:nlsTrpR)*	This manuscript	N/A
Danio rerio (zebrafish): *Tg(tUAS:SFiGluSnFR)*	This manuscript	N/A
*Paramecium caudatum*	Sciento	Cat#P320

**Recombinant DNA**		

pBH-opn1sw1-SyGCaMP6f-pA	This manuscript	N/A
pBH	Yoshimatsu et al. (2016)	N/A
p5E-opn1sw1	Yoshimatsu et al. (2016)	N/A
pME-SyGCaMP6f	This manuscript	N/A
p3E-pA	Kwan et al. (2007)	N/A
p5E-tUAS	Suli et al. (2014)	N/A
pME-SFiGluSnFR	This manuscript	N/A
pBH-tUAS-SFiGluSnFR-pA	This manuscript	N/A
pBH-cx55.5-nlsTrpR-pA	Yoshimatsu et al. (2016)	N/A

**Software and Algorithms**		

Igor 6 pro	Wavematrics	N/A
MATLAB	Mathworks	N/A
ImageJ	Schneider et al. (2012)	https://imagej.nih.gov/ij/
Phototransduction model algorithms	Invergo et al., 2013, Invergo et al., 2014	N/A

### Resource Availability

#### Lead Contact

Further information and requests for resources and reagents should be directed to and will be fulfilled by the Lead Contact, Tom Baden (t.baden@sussex.ac.uk).

#### Materials Availability

Plasmids pTo2pA-opn1sw1-SyGCaMP6f-pA, pBH-tUAS-SFiGluSnFR-pA, pME-SyGCaMP6f, pME-SFiGluSnFR, and transgenic lines *Tg(opn1sw1:GFP:SyGCaMP6f)*, *Tg(cx55.5:nlsTrpR)*, and *Tg(tUAS:SFiGluSnFR)* lines, generated in this study, are available upon request to the lead contact.

#### Data and Code Availability

Pre-processed functional 2-photon imaging data, cone-density counts, natural imaging data, transcriptome data, and associated summary statistics will be made freely available from DataDryad via the relevant links on https://datadryad.org/stash/landing/show?id=doi%3A10.5061%2Fdryad.w0vt4b8n3 and at https://www.badenlab.org/resources and http:/retinal-functomics.net/. Any remaining data will be provided upon reasonable request to the Lead Contact.

### Experimental Model and Subject Details

#### Animals

All procedures were performed in accordance with the UK Animals (Scientific Procedures) act 1986 and approved by the animal welfare committee of the University of Sussex. For all experiments, we used *6-8 days post fertilization* (*dpf*) zebrafish (Danio rerio) larvae. The following previously published transgenic lines were used: *Tg(opn1sw1:nfsBmCherry)* (Yoshimatsu et al., 2016), *Tg(opn1sw1:GFP)* (Takechi et al., 2003). In addition, *Tg(opn1sw1:GFP:SyGCaMP6f)*, *Tg(cx55.5:nlsTrpR)*, and *Tg(tUAS:SFiGluSnFR)* lines were generated by injecting pBH-opn1sw1-SyGCaMP6f-pA, pBH-cx55.5-nlsTrpR-pA (Yoshimatsu et al., 2016), or pBH-tUAS-SFiGluSnFR-pA plasmids into single-cell stage eggs. Injected fish were out-crossed with wild-type fish to screen for founders. Positive progenies were raised to establish transgenic lines.

All plasmids were made using the Gateway system (ThermoFisher, 12538120) with combinations of entry and destination plasmids as follows: pBH-opn1sw1-SyGCaMP6f-pA: pBH and p5E-opn1sw1 (Yoshimatsu et al., 2016), pME-SyGCaMP6f, p3E-pA (Kwan et al., 2007); pBH-tUAS-SFiGluSnFR-pA: pBH (Yoshimatsu et al., 2016), p5E-tUAS (Suli et al., 2014), pME-SFiGluSnFR, p3E-pA. Plasmid pME-SyGCaMP6f was generated by inserting a polymerase chain reaction (PCR)-amplified GCaMP6f (Chen et al., 2013) into pME plasmid and subsequently inserting a PCR amplified zebrafish synaptophysin without stop codon at the 5′ end of GCaMP6f. pME-SFiGluSnFR was made by inserting a PCR amplified SFiGluSnFR (Marvin et al., 2018) fragment in pME plasmid.

Animals were housed under a standard 14:10 day/night rhythm and fed three times a day. Animals were grown in 0.1 mM 1-phenyl-2-thiourea (Sigma, P7629) from 1 *dpf* to prevent melanogenesis. For 2-photon *in-vivo* imaging, zebrafish larvae were immobilised in 2% low melting point agarose (Fisher Scientific, BP1360-100), placed on a glass coverslip and submerged in fish water. Eye movements were prevented by injection of a-bungarotoxin (1 nL of 2 mg/ml; Tocris, Cat: 2133) into the ocular muscles behind the eye. For some experiments, CNQX (∼0.5 pl, 2 mM, Tocris, Cat: 1045) in artificial cerebro-spinal fluid (aCSF) was injected into the eye.

### Method Details

#### Imaging the appearance of paramecia at different wavelengths of light

*Paramecium caudatum* (Sciento, P320) were placed in a container filled with fish water and pebbles, to approximately mimic a zebrafish natural habitat (Zimmermann et al., 2018). Images were taken outdoors under the sun (typical sunny day in UK, Brighton in May, no cloud at around 1 pm) with a CCD camera (Thorlabs DCU223M) fitted with a lens (Thorlabs ACL1815L), a constitutive glass filter (Thorlabs FGB37) as well as switchable glass filters (UV: FGUV11-UV, Yellow: FGV9; both Thorlabs) on a filter-wheel. Videos were acquired at 10 Hz, with single frame exposure times of 1 and 70 ms for yellow and UV, respectively. The focal distance of the camera was ∼2.5 cm, and it was positioned against the wall of the tank from the outside. The effective recording spectra were computed by multiplying the spectral sensitivity of the camera chip itself with all optical components in the path.

#### Behavioral experiments

Individual 7-8 *dpf* zebrafish larva were head-mounted in 2% low-melting-point agarose (Fisher Scientific, BP1360-100) in a 35 mm Petri dish with the eyes and tail free to move and filmed under infrared illumination (940 nm) using a Raspberry Pi camera at 30 Hz based on a previous design (Maia Chagas et al., 2017). An Arduino-microcontroller was used to iteratively switch top-illumination of the dish between UV (374 ± 15 nm) or yellow (507 ± 10 nm) LED light in periods of 1 minute. The peak power of both LEDs was set to be equal at 0.12 W m^-2^. The same fish was filmed continuously for three such cycles (total of 12 minutes per n = 12 fish wild-type and another n = 12 fish with UV cones ablated), and behavioral performance was manually annotated offline as either a “full prey capture bout” (eye convergence plus tail movement) or “tracking” (single or bilateral eye movements in the absence of tail movements). To ablate UV-cones, *Tg(opn1sw1:nfsBmCherry)* larval zebrafish were treated with 10 mM Metronidazole (Sigma, M3761) for 2 hours and thereafter transferred to fresh fish water without Metronidazole. Behavioral assays were performed one day after the Metronidazole treatment to ensure that UV-cone ablation was complete (Yoshimatsu et al., 2016).

#### UV-cone density estimation across the visual field

The UV-cone distribution across the eye was first established from confocal image stacks of Tg(opn1sw1:GFP) eyes from *7 dpf* larvae where all UV-cones are labeled. Fish were mounted with one eye facing the objective lens. As in previous work (Zimmermann et al., 2018) the locations of all UV-cones in the 3D eye were detected using a custom script in Igor Pro 6.3 (Wavemetrics). To project the resultant UV-cone distribution into visual space, we first measured the eye size as being 300 μm on average. In addition, we determined that both the eyeball and the lens follow a nearly perfect spherical curvature with a common point of origin. From this, we assumed that any given UV-cone collects light from a point in the space that aligns with a straight line connecting the UV-cone to the outside world through the center of the lens. From here, we mapped UV-cone receptive field locations across the full monocular visual field.

#### Immunostaining, dye-staining and confocal imaging

Larval zebrafish (*7-8 dpf*) were euthanised by tricane overdose and then fixed in 4% paraformaldehyde (PFA, Agar Scientific, AGR1026) in PBS for 30 min at room temperature. After three washes in PBS, whole eyes were enucleated and the cornea was removed by hand using the tip of a 30 G needle. Dissected and fixed samples were treated with PBS containing 0.5% Triton X-100 (Sigma, X100) for at least 10 mins and up to 1 day, followed by the addition of primary antibodies. After 3-5 days incubation at 4°C, samples were washed three times with PBS 0.5% Triton X-100 solution and treated with secondary antibodies and/or BODIPY (Invitrogen, C34556) dye. After one day incubation, samples were mounted in 1% agar in PBS on a coverslip and subsequently PBS was replaced with mounting media (VectaShield, H-1000) for imaging. Primary antibodies used were anti-GFP (abcom, chicken, ab13970) and anti-CoxIV (abcom, rabbit, ab209727). Secondary antibodies were Donkey CF488A dye anti-chick (Sigma, SAB4600031) and Goat Alexa647 dye anti-rabbit (ThermoFisher, A-21244). Confocal image stacks were taken on a TSC SP8 (Leica) with 40x water immersion objective (C PL APO CS2, Leica), a 63x oil immersion objective (HC PL APO CS2, Leica) or a 20x dry objective (HC PL APO Dry CS2, Leica). Typical voxel size was 150 nm and 1 μm in xy and z, respectively. Contrast, brightness and pseudo-color were adjusted for display in Fiji (NIH). Quantification of outer segment lengths and anti-GFP staining intensity was performed using custom scripts in Igor Pro 6.3 (Wavemetrics) after manually marking outer segment outer and inner locations.

#### 2-photon calcium and glutamate imaging and light stimulation

All 2-photon imaging was performed on a MOM-type 2-photon microscope (designed by W. Denk, MPI, Martinsried; purchased through Sutter Instruments/Science Products) equipped with a mode-locked Ti:Sapphire laser (Chameleon Vision-S, Coherent) tuned to 927 or 960 nm for SyGCaMP6f and SFiGluSnFR imaging and 960 nm for mCherry and SFiGluSnFR double imaging. We used two fluorescence detection channels for SyGCaMP6f/iGluRSnFR (F48x573, AHF/Chroma) and mCherry (F39x628, AHF/Chroma), and a water immersion objective (W Plan-Apochromat 20x/1,0 DIC M27, Zeiss). For image acquisition, we used custom-written software (ScanM, by M. Mueller, MPI, Martinsried and T. Euler, CIN, Tuebingen) running under IGOR pro 6.3 for Windows (Wavemetrics). Recording configurations were as follows: SyGCaMP6f UV flashes Figures 3 and 4: 128x16 pixels (1 ms per line, 62.5 Hz); SyGCaMP6f whole-eye Figure 6: 512x512 pixels (2 ms per line, 0.97 Hz), SFiGluSnFR noise recording Figures 8D–8G: 128x32 pixels (1 ms per line, 31.25 Hz), SFiGluSnFR and SyGCaMP6f noise recordings Figure 8H: 64x4 pixels (2 ms per line, 125 Hz). Light stimulation was setup-up as described previously (Zimmermann et al., 2018, Zimmermann et al., 2020). In brief, light stimuli were delivered through the objective, by band-pass filtered light emitting diodes (LEDs) (‘red’ 588 nm, B5B-434-TY, 13.5cd, 20 mA; ‘green’ 477 nm, RLS-5B475-S; 3-4 cd, 20mA; ‘blue’ 415 nm, VL415-5-15; 10-16 mW, 20 mA; ‘ultraviolet, UV’ 365 nm, LED365-06Z; 5.5 mW, 20 mA, Roithner, Germany). LEDs were filtered and combined using FF01-370/36, T450/pxr, ET420/40 m, T400LP, ET480/40x, H560LPXR (AHF/Chroma) and synchronized with the scan retrace at 500 (2 ms lines) or 1,000 Hz (1 ms lines) using a microcontroller and custom scripts (available at https://github.com/BadenLab/Zebrafish-visual-space-model). The ratio of LED intensities was calibrated (in photons per s per cone) such that each LED would relatively stimulate its respective cone-type as it would be activated under natural spectrum light in the zebrafish habitat (Zimmermann et al., 2018): 34, 18, 4.7 and 2.1 x10^5^ photons per cone per s for red-, green-, blue-, and UV-cones, respectively. We used these “natural spectrum” LED intensities as a background light and modulated contrasts depends on experiments. LED contrasts were 0% for dark and 2,500% for bright flashes (Figures 3B–3F), 0% background and 2,500% flash (Figures 3G and 3H), 2,500% background and 0% dark flash (Figures 4A and 4C), 0% dark and 200% bright (Figure 6). For tetrachromatic noise (Figures 7 and 8), each of 4 LEDs was simultaneously but independently presented at 100% contrast in a known sequence at 12.8 Hz. Short 5 ms UV flashes with intensities spanning from 67 to 10^4^ photons/cone were delivered to measure UV-cone sensitivities (Figures 3I and 3L) and light-recovery kinetics (Figures 4E and 4F). For all experiments, the animal was kept at constant background illumination for at least 5 s at the beginning of each recording to allow for adaptation to the laser.

#### UV-cone activation model

Cone distributions were taken from published data (Zimmermann et al., 2018). UV- and blue-cones were taken from the same representative eye and aligned with red- and green-cones from a second eye and projected into visual space. The full array was cropped at ± 60°. Model BCs were randomly spaced at a minimum radius of 10°. BCs summed the activity from all cones within this same fixed radius. Target trajectory was computed as a random walk on an infinite plane (canonical diffeomorphism), such as the left/right and top/bottom borders are continuous with each other. At each 1° step-size iteration (equivalent to 10 ms), the target advanced at a constant speed of 100°/s with a random change of angle (α) that satisfied −15° < α < 15°. Cone activation by the moving target was computed as follows: At each time-point, the distance between the centers of the target and each cone was determined. If this distance was smaller than the sum of the target radius (1° and 2.5° for light and dark target, respectively) and a cone’s receptive field radius (0.38°), the cone was activated to yield a binary activation sequence over time for each cone. This sequence was then convolved with the cone’s impulse response. Here, the peak amplitude and recovery time constant was assigned based on a cone’s position, drawing on the four measurement points established from calcium imaging (dorsal, nasal i.e., horizon, ventral and SZ, *cf.* Figure 3). Along the dorsal-ventral axis, values were chosen based on the relative distance between the horizon and the dorsal or ventral edge. For example, a cone positioned 75% toward the dorsal edge from the horizon would be assigned values weighted as 0.75:0.25:0 from dorsal, nasal and ventral measurements, respectively. In addition, if a cone was within 30° of the SZ center (−30°,-30°), it was in addition weighted based on values from the SZ in the same way. In each run, all activation values were normalized to the peak activation across the entire array.

#### RNA-sequencing of UV-cones

Whole *7 dpf* Tg(*opn1sw1:GFP*) larval zebrafish retinas were dissected in carboxygenated aCSF (CaCl 0.1275 g/L, MgSO_4_ 0.1488 g/L, KCl 0.231 g/L, KH_2_PO_4_ 0.068 g/L, NaCl 7.01 g/L, D-Glucose 1.081 g/L, and NaHCO_3_ 1.9 g/L) while keeping track of each retina’s orientation. Each retina was then cut into two pieces: SZ, and non-SZ. Typically tissues from ∼10 fish (20 eyes) were batched into one tube and dissociated using a papain dissociation system (Worthingtonm LK003176, LK003170, LK003182) with the following modification in the protocol: Incubation in papain for 10 min at room temperature. During dissociation, tissues were gently pipetted every 3 min to facilitate dissociation using glass pipette with rounded tip. After 10 min incubation, DNase and ovomucoid were added and the tissues were further mechanically dissociated by gentle pipetting. Dissociated cells were immediately sorted for GFP expression by FACSMelody (BD Biosciences). Approximately 100 cells were sorted in one tube, flash frozen in liquid nitrogen and stored at −80 degree until further use. Libraries were prepared using Ultra-low input RNA kit (Takara, 634888) and subjected to next generation sequencing at GENEWIZ (NZ, US). Sequencing data was quality checked and trimmed to remove adaptors using Trim Galore!([CSL STYLE ERROR: reference with no printed form.]), aligned on the zebrafish genome (GRCz11.9) in HISAT2 (Kim et al., 2015), and counted for gene expression in featureCounts (Liao et al., 2014) using the public server at the usergalaxy.org online platform (Afgan et al., 2018). In total, four repeats each were performed for SZ and non-SZ samples.

#### Differential gene expression analysis

For the analysis of differential gene expression of the SZ versus non-SZ we used the DESeq2 package in R/Bioconductor (Love et al., 2014). We only included genes which had a count of at least 5 sequence fragments in at least 2 of the 8 samples (4 SZ + 4 non-SZ). Since we wanted to measure the effect between zones, controlling for differences in the individual eyes, we included the eye as an additional latent variable (design = ∼eye+zone). The DESeq2 package then uses a generalized linear model with a logarithmic link to infer a negative-binomial distribution for gene counts (Love et al., 2014). The inferred means via the *poscount* estimator, which calculates a modified geometric mean by taking the n^th^ root of the product of non-zero counts, are shown in Figures 7B and 7C. The log-fold changes (Figure 7D) were then also estimated in DESeq2.

For determining differential expression normalized by UV-opsin (Figure 7E) we instead calculated using the raw count data, normalized by the count of the UV-opsin gene. From here, mean fold changes were calculated by taking fold changes of individual SZ and non-SZ sample pairs.

#### Modeling phototransduction

We used a previously described and verified computational model of phototransduction in vertebrate ciliary photoreceptors (Invergo et al., 2013, Invergo et al., 2014). We simulated the photo-response to 100% dark or 100% bright contrasts (Figures 7G and 7H) or to 5 ms flashes from dark of various intensities (Figures 7J and 7M) using default parameters provided by the model for non-SZ simulation. For simulating the SZ, we then scaled all according to the relative gene expression change between SZ and nSZ conditions. Transducin was scaled by taking the lowest value among components (gngt2b, gnb3b, gnat2) because all components are necessary for transducin function. Similarly, we scaled CNG based on the CNGa3 expression level. Parameters changed for each condition are listed in Table S4.

#### Software

Data analysis was performed using IGOR Pro 6.3 (Wavemetrics), Fiji (NIH), Python 3.5 (Anaconda distribution, scikit-learn 0.18.1, scipy 0.19.0 and pandas 0.20.1) and R 3.5.1.

#### Pre-processing and Dark-Light-index

Regions of interest (ROIs), corresponding to individual presynaptic terminals of UV-cones were defined automatically based on local thresholding of the recording stack’s s.d. projection over time (s.d. typically > 25), followed by filtering for size and shape using custom written software on IGOR Pro 6.3 (Wavemetrics). Specifically, only round ROIs (< 150% elongation) of size 2-5 μm^2^ were further analyzed. For glutamate recording, ROIs were manually placed as the shape of HC dendritic terminals at cone terminals are often skewed. Calcium or glutamate traces for each ROI were extracted and z-normalized based on the time interval 1-6 s at the beginning of recordings prior to presentation of systematic light stimulation. A stimulus time marker embedded in the recording data served to align the traces relative to the visual stimulus with a temporal precision of 1 or 2 ms (depending on line-scan speed). The Dark-Light-index (DLi) was calculated as:

$$DLi=\frac{L-D}{L+D}$$

where *L* and *D* are the mode of response amplitudes to UV- and dark-flash with RGB background, respectively.

#### Information Rates

To calculate information rates, we first filtered recorded traces for quality: We calculated the linear response kernel to UV-light stimulation for each trace and took only the traces where the response amplitude of the kernel, measured as its standard deviation, was at least 70% of the kernel with maximal response amplitude of the same zone.

We then followed the procedure as described in ref (van Hateren and Snippe, 2001) using the bias correction method for finite data. For this, we assumed that the noise between repetitions of the experiment was statistically independent. For independent Gaussian statistics, the information rate *R* can be computed as:

$$R=\int_{0}^{\infty }\log_{2}\left(1+SNR\left(f\right)\right)df.$$

Since photoreceptors are best driven by low frequency signals (Baden et al., 2013a) we chose a cut-off frequency of 12 Hz. We then calculated a bias corrected signal to noise ratio (SNR) as:

$$S\left(t\right)=\frac{1}{n}\sum_{i}X_{i}\left(t\right)\;N\left(t\right)=\frac{1}{n}\sum_{i}\left(S\left(t\right)-X_{i}\left(t\right)\right)$$

$$SNR\left(f\right)=\frac{1}{n-1}\cdot \frac{\hat{S}\left(f\right)}{\hat{N}\left(f\right)}-\frac{1}{n}$$

where *X*_*i*_ is an individual trial, *n* is the number of trials and $\hat{S}$ and $\hat{N}$ are the Fourier transform of *S* and *N,* respectively. We used Welch’s method to reduce noise in the estimated power spectra.

### Quantification and Statistical Analysis

#### Statistics

No statistical methods were used to predetermine sample size. P values were calculated using non-parametric Mann-Whitney U tests in Figures 1H and S1G, and using a paired t test in Figure 1G. For Figures 4B and 4D–4F, p values were calculated using ANOVA with factors treatment and area interaction, and posthoc tests with tukey correction for multiple testing. The posthoc tables are provided in Tables S1–S3 and only stars for relevant comparisons are added to the figures. Owing to the exploratory nature of our study, we did not use randomization or blinding.

We used Generalized Additive Models (GAMs) to analyze the relationships between eye position and outer segment size, baseline, and dark-light index (Figures 2G, 6C, 6E, 6G, and S2E). GAMs can be seen as an extension to the generalized linear model by allowing linear predictors, which depend on smooth functions of the underlying variables (Wood, 2006). We used the mgcv-package (version 1.8-28) on a Windows 10 workstation (8 Xeon E3-1270 v5 3.6 GHz; 64 GB RAM) with default parameters. We modeled the dependence of the variable of interest as a smooth term with 20 degrees of freedom. In addition, we incorporated the fish id as a random effect. The models explained ∼40%–80% of the deviance. For plotting, we generated the predicted mean response with approximate 95% confidence intervals excluding fish id (this leads to a slight perceived offset between the raw data points and the mean response). Statistical significance for differences between the dependence of DLi in baseline and HC block conditions were obtained using the plot_diff function of the itsadug-package for R (version 2.3).

## Supplemental Information

Document S1. Figures S1–S3 and Tables S1–S4

Document S2. Article plus Supplemental Information

## References

[bib1] Afgan E., Baker D., Batut B., van den Beek M., Bouvier D., Čech M., Chilton J., Clements D., Coraor N., Grüning B.A. (2018). The Galaxy platform for accessible, reproducible and collaborative biomedical analyses: 2018 update. Nucleic Acids Res..

[bib2] Ala-Laurila P., Greschner M., Chichilnisky E.J., Rieke F. (2011). Cone photoreceptor contributions to noise and correlations in the retinal output. Nat. Neurosci..

[bib3] Angueyra J.M., Rieke F. (2013). Origin and effect of phototransduction noise in primate cone photoreceptors. Nat. Neurosci..

[bib4] Armbruster M., Dulla C.G., Diamond J.S. (2019). Effects of fluorescent glutamate indicators on neurotransmitter diffusion and uptake. bioRxiv.

[bib5] Baden T., Osorio D. (2019). The retinal basis of vertebrate color vision. Annu. Rev. Vis. Sci..

[bib6] Baden T., Euler T., Weckström M., Lagnado L. (2013). Spikes and ribbon synapses in early vision. Trends Neurosci..

[bib7] Baden T., Schubert T., Chang L., Wei T., Zaichuk M., Wissinger B., Euler T. (2013). A tale of two retinal domains: near-optimal sampling of achromatic contrasts in natural scenes through asymmetric photoreceptor distribution. Neuron.

[bib8] Baden T., Euler T., Berens P. (2019). Retinal circuits for vision across species. Nat. Rev. Neurosci..

[bib9] Baudin J., Angueyra J.M., Sinha R., Rieke F. (2019). S-cone photoreceptors in the primate retina are functionally distinct from L and M cones. eLife.

[bib10] Behrens C., Schubert T., Haverkamp S., Euler T., Berens P., Baden T., Schubert T., Chang L., Wei T., Zaichuk M. (2016). Connectivity map of bipolar cells and photoreceptors in the mouse retina. eLife.

[bib11] Bellono N.W., Leitch D.B., Julius D. (2018). Molecular tuning of electroreception in sharks and skates. Nature.

[bib12] Bianco I.H., Kampff A.R., Engert F. (2011). Prey capture behavior evoked by simple visual stimuli in larval zebrafish. Front. Syst. Neurosci..

[bib13] Bleckert A., Schwartz G.W., Turner M.H., Rieke F., Wong R.O.L. (2014). Visual space is represented by nonmatching topographies of distinct mouse retinal ganglion cell types. Curr. Biol..

[bib14] Branchek T., Bremiller R. (1984). The development of photoreceptors in the zebrafish, Brachydanio rerio. I. Structure. J. Comp. Neurol..

[bib15] Bringmann A. (2019). Structure and function of the bird fovea. Anat. Histol. Embryol..

[bib16] Bringmann A., Syrbe S., Görner K., Kacza J., Francke M., Wiedemann P., Reichenbach A. (2018). The primate fovea: Structure, function and development. Prog. Retin. Eye Res..

[bib17] Chapot C.A., Behrens C., Rogerson L.E., Baden T., Pop S., Berens P., Euler T., Schubert T. (2017). Local signals in mouse horizontal cell dendrites. Curr. Biol..

[bib18] Chapot C.A., Euler T., Schubert T. (2017). How do horizontal cells ‘talk’ to cone photoreceptors? Different levels of complexity at the cone-horizontal cell synapse. J. Physiol..

[bib19] Chen T.-W., Wardill T.J., Sun Y., Pulver S.R., Renninger S.L., Baohan A., Schreiter E.R., Kerr R.A., Orger M.B., Jayaraman V. (2013). Ultrasensitive fluorescent proteins for imaging neuronal activity. Nature.

[bib20] Chinen A., Hamaoka T., Yamada Y., Kawamura S. (2003). Gene duplication and spectral diversification of cone visual pigments of zebrafish. Genetics.

[bib21] Collin S.P., Lloyd D.J., Wagner H.-J. (2000). Foveate vision in deep-sea teleosts: a comparison of primary visual and olfactory inputs. Phil. Trans. R. Soc. Lond. B.

[bib22] Connaughton V.P., Maguire G. (1998). Differential expression of voltage-gated K+ and Ca2+ currents in bipolar cells in the zebrafish retinal slice. Eur. J. Neurosci..

[bib23] Connaughton V.P., Nelson R. (2000). Axonal stratification patterns and glutamate-gated conductance mechanisms in zebrafish retinal bipolar cells. J. Physiol..

[bib24] Connaughton V.P., Nelson R. (2015). Ultraviolet dominates ganglion cell responses in larval zebrafish. Invest. Ophthalmol. Vis. Sci..

[bib25] Cronin T.W., Bok M.J. (2016). Photoreception and vision in the ultraviolet. J. Exp. Biol..

[bib26] Cronin T.W., Johnsen S., Marshall N.J., Warrant E.J. (2014). Visual Ecology.

[bib27] Curcio C.A., Sloan K.R., Kalina R.E., Hendrickson A.E. (1990). Human photoreceptor topography. J. Comp. Neurol..

[bib28] de Busserolles F., Fitzpatrick J.L., Marshall N.J., Collin S.P. (2014). The influence of photoreceptor size and distribution on optical sensitivity in the eyes of lanternfishes (Myctophidae). PLoS ONE.

[bib29] Dizhoor A., Ray S., Kumar S., Niemi G., Spencer M., Brolley D., Walsh K., Philipov P., Hurley J., Stryer L. (1991). ). Recoverin: a calcium sensitive activator of retinal rod guanylate cyclase. Science.

[bib30] Dräger U.C., Olsen J.F. (1981). Ganglion cell distribution in the retina of the mouse. Invest. Ophthalmol. Vis. Sci..

[bib31] Dreosti E., Odermatt B., Dorostkar M.M., Lagnado L. (2009). A genetically encoded reporter of synaptic activity in vivo. Nat. Methods.

[bib32] Dreosti E., Esposti F., Baden T., Lagnado L. (2011). In vivo evidence that retinal bipolar cells generate spikes modulated by light. Nat. Neurosci..

[bib33] Dunn T.W.W., Gebhardt C., Naumann E.A.A., Riegler C., Ahrens M.B.B., Engert F., Del Bene F. (2016). Neural circuits underlying visually evoked escapes in larval zebrafish. Neuron.

[bib34] Enright J.M., Toomey M.B., Sato S.Y., Temple S.E., Allen J.R., Fujiwara R., Kramlinger V.M., Nagy L.D., Johnson K.M., Xiao Y. (2015). Cyp27c1 red-shifts the spectral sensitivity of photoreceptors by converting Vitamin A1 into A2. Curr. Biol..

[bib35] Esposti F., Johnston J., Rosa J.M., Leung K.-M., Lagnado L. (2013). Olfactory stimulation selectively modulates the OFF pathway in the retina of zebrafish. Neuron.

[bib36] Fain G.L., Hardie R., Laughlin S.B. (2010). Phototransduction and the evolution of photoreceptors. Curr. Biol..

[bib37] Field G.D., Sampath A.P., Rieke F. (2005). Retinal processing near absolute threshold: from behavior to mechanism. Annu. Rev. Physiol..

[bib38] Frank T., Khimich D., Neef A., Moser T. (2009). Mechanisms contributing to synaptic Ca2+ signals and their heterogeneity in hair cells. Proc. Natl. Acad. Sci. USA.

[bib39] Franke K., Berens P., Schubert T., Bethge M., Euler T., Baden T. (2017). Inhibition decorrelates visual feature representations in the inner retina. Nature.

[bib40] Franke K., Maia Chagas A., Zhao Z., Zimmermann M.J.Y., Bartel P., Qiu Y., Szatko K.P., Baden T., Euler T. (2019). An arbitrary-spectrum spatial visual stimulator for vision research. eLife.

[bib41] Gahtan E., Tanger P., Baier H. (2005). Visual prey capture in larval zebrafish is controlled by identified reticulospinal neurons downstream of the tectum. J. Neurosci..

[bib42] Giarmarco M.M., Cleghorn W.M., Sloat S.R., Hurley J.B., Brockerhoff S.E. (2017). Mitochondria maintain distinct Ca^2+^ pools in cone photoreceptors. J. Neurosci..

[bib43] Hardie R.C. (1984). Properties of photoreceptors R7 and R8 in dorsal marginal ommatidia in the compound eyes of musca and calliphora. J. Comp. Physiol. A.

[bib44] Haug M.F., Biehlmaier O., Mueller K.P., Neuhauss S.C. (2010). Visual acuity in larval zebrafish: behavior and histology. Front. Zool..

[bib45] Heath S.L., Christenson M.P., Oriol E., Saavedra-Weisenhaus M., Kohn J.R., Behnia R. (2020). Circuit mechanisms underlying chromatic encoding in Drosophila photoreceptors. Curr. Biol..

[bib46] Heidelberger R., Thoreson W.B., Witkovsky P. (2005). Synaptic transmission at retinal ribbon synapses. Prog. Retin. Eye Res..

[bib47] Hurley J.B. (1987). Molecular properties of the cGMP cascade of vertebrate photoreceptors. Annu. Rev. Physiol..

[bib48] Invergo B.M., Montanucci L., Koch K.-W., Bertranpetit J., Dell’orco D. (2013). Exploring the rate-limiting steps in visual phototransduction recovery by bottom-up kinetic modeling. Cell Commun. Signal..

[bib49] Invergo B.M., Dell’Orco D., Montanucci L., Koch K.-W., Bertranpetit J. (2014). A comprehensive model of the phototransduction cascade in mouse rod cells. Mol. Biosyst..

[bib50] James B., Darnet L., Moya-Díaz J., Seibel S.-H., Lagnado L. (2019). An amplitude code transmits information at a visual synapse. Nat. Neurosci..

[bib51] Johnsen S., Widder E.A. (2001). Ultraviolet absorption in transparent zooplankton and its implications for depth distribution and visual predation. Mar. Biol..

[bib52] Jouary A., Haudrechy M., Candelier R., Sumbre G. (2016). A 2D virtual reality system for visual goal-driven navigation in zebrafish larvae. Sci. Rep..

[bib53] Jung I., Powers T.R., Valles J.M. (2014). Evidence for two extremes of ciliary motor response in a single swimming microorganism. Biophys. J..

[bib54] Kamermans M., Kraaij D., Spekreijse H. (2001). The dynamic characteristics of the feedback signal from horizontal cells to cones in the goldfish retina. J. Physiol..

[bib55] Kemp C.M., Faulkner D.J., Jacobson S.G. (1988). The distribution and kinetics of visual pigments in the cat retina. Invest. Ophthalmol. Vis. Sci..

[bib56] Kim D., Langmead B., Salzberg S.L. (2015). HISAT: a fast spliced aligner with low memory requirements. Nat. Methods.

[bib57] Klaassen L.J., Fahrenfort I., Kamermans M. (2012). Connexin hemichannel mediated ephaptic inhibition in the retina. Brain Res..

[bib58] Klaassen L.J., de Graaff W., van Asselt J.B., Klooster J., Kamermans M. (2016). Specific connectivity between photoreceptors and horizontal cells in the zebrafish retina. J. Neurophysiol..

[bib59] Knabe W., Skatchkov S., Kuhn H.-J. (1997). “Lens mitochondria” in the retinal cones of the tree-shrew Tupaia belangeri. Vision Res..

[bib60] Kwan K.M., Fujimoto E., Grabher C., Mangum B.D., Hardy M.E., Campbell D.S., Parant J.M., Yost H.J., Kanki J.P., Chien C.-B. (2007). The Tol2kit: a multisite gateway-based construction kit for Tol2 transposon transgenesis constructs. Dev. Dyn..

[bib61] Lagnado L., Schmitz F. (2015). Ribbon synapses and visual processing in the retina. Annu. Rev. Vis. Sci..

[bib62] Lamb T.D. (2013). Evolution of phototransduction, vertebrate photoreceptors and retina. Prog. Retin. Eye Res..

[bib63] Lamb T.D. (2016). Why rods and cones?. Eye (Lond.).

[bib64] Lamb T.D., Collin S.P., Pugh E.N. (2007). Evolution of the vertebrate eye: opsins, photoreceptors, retina and eye cup. Nat. Rev. Neurosci..

[bib65] Land M.F. (2015). Eye movements of vertebrates and their relation to eye form and function. J. Comp. Physiol. A Neuroethol. Sens. Neural Behav. Physiol..

[bib66] Land M.F., Nilsson D.-E. (2012). Animal Eyes.

[bib67] Lawrence C. (2007). The husbandry of zebrafish (Danio rerio): a review. Aquaculture.

[bib68] Li Y.N., Matsui J.I., Dowling J.E. (2009). Specificity of the horizontal cell-photoreceptor connections in the zebrafish (Danio rerio) retina. J. Comp. Neurol..

[bib69] Li Y.N., Tsujimura T., Kawamura S., Dowling J.E. (2012). Bipolar cell-photoreceptor connectivity in the zebrafish (Danio rerio) retina. J. Comp. Neurol..

[bib70] Liao Y., Smyth G.K., Shi W. (2014). featureCounts: an efficient general purpose program for assigning sequence reads to genomic features. Bioinformatics.

[bib71] Losey G.S., Cronin T.W.T., Goldsmith T.H., Hydes D., Marshall N.J.N., McFarland W.N., Hyde D., Marshall N.J.N., McFarland W.N. (1999). The UV visual world of fishes: A review. J. Fish Biol..

[bib72] Love M.I., Huber W., Anders S. (2014). Moderated estimation of fold change and dispersion for RNA-seq data with DESeq2. Genome Biol..

[bib73] Maia Chagas A., Prieto-Godino L.L., Arrenberg A.B., Baden T. (2017). The €100 lab: a 3D-printable open-source platform for fluorescence microscopy, optogenetics, and accurate temperature control during behaviour of zebrafish, Drosophila, and Caenorhabditis elegans. PLoS Biol..

[bib74] Marvin J.S., Scholl B., Wilson D.E., Podgorski K., Kazemipour A., Müller J.A., Schoch S., Quiroz F.J.U., Rebola N., Bao H. (2018). Stability, affinity, and chromatic variants of the glutamate sensor iGluSnFR. Nat. Methods.

[bib75] Masland R.H. (2001). The fundamental plan of the retina. Nat. Neurosci..

[bib76] McElligott M.B., O’malley D.M. (2005). Prey tracking by larval zebrafish: axial kinematics and visual control. Brain Behav. Evol..

[bib77] Mearns D.S., Donovan J.C., Fernandes A.M., Semmelhack J.L., Baier H. (2020). Deconstructing hunting behavior reveals a tightly coupled stimulus-response Loop. Curr. Biol..

[bib78] Moser T., Grabner C.P., Schmitz F. (2020). Sensory processing at ribbon synapses in the retina and the cochlea. Physiol. Rev..

[bib79] Mowat F.M., Petersen-Jones S.M., Williamson H., Williams D.L., Luthert P.J., Ali R.R., Bainbridge J.W. (2008). Topographical characterization of cone photoreceptors and the area centralis of the canine retina. Mol. Vis..

[bib80] Muto A., Kawakami K. (2013). Prey capture in zebrafish larvae serves as a model to study cognitive functions. Front. Neural Circuits.

[bib81] Muto A., Ohkura M., Abe G., Nakai J., Kawakami K. (2013). Real-time visualization of neuronal activity during perception. Curr. Biol..

[bib82] Nathans J. (1999). The evolution and physiology of human color vision: insights from molecular genetic studies of visual pigments. Neuron.

[bib83] Novales Flamarique I. (2012). Opsin switch reveals function of the ultraviolet cone in fish foraging. Proc. Biol. Sci..

[bib84] Novales Flamarique I. (2016). Diminished foraging performance of a mutant zebrafish with reduced population of ultraviolet cones. Proc. Biol. Sci..

[bib85] Okawa H., Sampath A.P., Laughlin S.B., Fain G.L. (2008). ATP consumption by mammalian rod photoreceptors in darkness and in light. Curr. Biol..

[bib86] Packer O., Hendrickson A.E., Curcio C.A. (1989). Photoreceptor topography of the retina in the adult pigtail macaque (Macaca nemestrina). J. Comp. Neurol..

[bib87] Patterson B.W., Abraham A.O., MacIver M.A., McLean D.L. (2013). Visually guided gradation of prey capture movements in larval zebrafish. J. Exp. Biol..

[bib88] Peng Y.-R., Shekhar K., Yan W., Herrmann D., Sappington A., Bryman G.S., van Zyl T., Do M.T.H., Regev A., Sanes J.R. (2019). Molecular classification and comparative taxonomics of foveal and peripheral cells in primate retina. Cell.

[bib89] Pergner J., Kozmik Z. (2017). Amphioxus photoreceptors - insights into the evolution of vertebrate opsins, vision and circadian rhythmicity. Int. J. Dev. Biol..

[bib90] Preuss S.J., Trivedi C.A., vom Berg-Maurer C.M., Ryu S., Bollmann J.H. (2014). Classification of object size in retinotectal microcircuits. Curr. Biol..

[bib91] Pugh E.N., Lamb T.D. (1993). Amplification and kinetics of the activation steps in phototransduction. Biochim. Biophys. Acta.

[bib92] Pugh E.N., Nikonov S., Lamb T.D. (1999). Molecular mechanisms of vertebrate photoreceptor light adaptation. Curr. Opin. Neurobiol..

[bib93] Regus-Leidig H., Brandstätter J.H. (2012). Structure and function of a complex sensory synapse. Acta Physiol. (Oxf.).

[bib94] Robinson J., Schmitt E.A., Hárosi F.I., Reece R.J., Dowling J.E. (1993). Zebrafish ultraviolet visual pigment: absorption spectrum, sequence, and localization. Proc. Natl. Acad. Sci. USA.

[bib95] Robles E., Laurell E., Baier H. (2014). The retinal projectome reveals brain-area-specific visual representations generated by ganglion cell diversity. Curr. Biol..

[bib96] Salinas-Navarro M., Jiménez-López M., Valiente-Soriano F.J., Alarcón-Martínez L., Avilés-Trigueros M., Mayor S., Holmes T., Lund R.D., Villegas-Pérez M.P., Vidal-Sanz M. (2009). Retinal ganglion cell population in adult albino and pigmented mice: a computerized analysis of the entire population and its spatial distribution. Vision Res..

[bib97] Sancer G., Kind E., Plazaola-Sasieta H., Balke J., Pham T., Hasan A., Münch L.O., Courgeon M., Mathejczyk T.F., Wernet M.F. (2019). Modality-specific circuits for skylight orientation in the fly visual system. Curr. Biol..

[bib98] Schmitt E.A., Dowling J.E. (1999). Early retinal development in the zebrafish, Danio rerio: light and electron microscopic analyses. J. Comp. Neurol..

[bib99] Schnaitmann C., Haikala V., Abraham E., Oberhauser V., Thestrup T., Griesbeck O., Reiff D.F. (2018). Color processing in the early visual system of Drosophila. Cell.

[bib131] Schneider C.A., Rasband W.S., Eliceiri K.W. (2012). NIH Image to ImageJ: 25 years of image analysis. Nat. Methods.

[bib100] Semmelhack J.L., Donovan J.C., Thiele T.R., Kuehn E., Laurell E., Baier H. (2014). A dedicated visual pathway for prey detection in larval zebrafish. eLife.

[bib101] Shaner N.C., Campbell R.E., Steinbach P.A., Giepmans B.N.G., Palmer A.E., Tsien R.Y. (2004). Improved monomeric red, orange and yellow fluorescent proteins derived from Discosoma sp. red fluorescent protein. Nat. Biotechnol..

[bib102] Shourav M.K., Kim J.K. (2017). Long-term tracking of free-swimming Paramecium caudatum in viscous media using a curved sample chamber. Micromachines (Basel).

[bib103] Sinha R., Hoon M., Baudin J., Okawa H., Wong R.O.L., Rieke F. (2017). Cellular and circuit mechanisms shaping the perceptual properties of the primate fovea. Cell.

[bib104] Spence R., Gerlach G., Lawrence C., Smith C. (2008). The behaviour and ecology of the zebrafish, Danio rerio. Biol. Rev. Camb. Philos. Soc..

[bib105] Stark R., Grzelak M., Hadfield J. (2019). RNA sequencing: the teenage years. Nat. Rev. Genet..

[bib106] Sterling P., Matthews G. (2005). Structure and function of ribbon synapses. Trends Neurosci..

[bib107] Suli A., Guler A.D., Raible D.W., Kimelman D. (2014). A targeted gene expression system using the tryptophan repressor in zebrafish shows no silencing in subsequent generations. Development.

[bib108] Suliman T., Novales Flamarique I. (2014). Visual pigments and opsin expression in the juveniles of three species of fish (rainbow trout, zebrafish, and killifish) following prolonged exposure to thyroid hormone or retinoic acid. J. Comp. Neurol..

[bib109] Szél A., Lukáts A., Fekete T., Szepessy Z., Röhlich P. (2000). Photoreceptor distribution in the retinas of subprimate mammals. J. Opt. Soc. Am. A Opt. Image Sci. Vis..

[bib110] Takechi M., Hamaoka T., Kawamura S. (2003). Fluorescence visualization of ultraviolet-sensitive cone photoreceptor development in living zebrafish. FEBS Lett..

[bib111] Thoreson W.B. (2007). Kinetics of synaptic transmission at ribbon synapses of rods and cones. Mol. Neurobiol..

[bib112] Thoreson W.B., Mangel S.C. (2012). Lateral interactions in the outer retina. Prog. Retin. Eye Res..

[bib113] Trivedi C.A., Bollmann J.H. (2013). Visually driven chaining of elementary swim patterns into a goal-directed motor sequence: a virtual reality study of zebrafish prey capture. Front. Neural Circuits.

[bib114] van Hateren J.H., Snippe H.P. (2001). Information theoretical evaluation of parametric models of gain control in blowfly photoreceptor cells. Vision Res..

[bib115] van Hateren J.H., van der Schaaf A. (1998). Independent component filters of natural images compared with simple cells in primary visual cortex. Proc. Biol. Sci..

[bib116] Van Hook M.J., Nawy S., Thoreson W.B. (2019). Voltage- and calcium-gated ion channels of neurons in the vertebrate retina. Prog. Retin. Eye Res..

[bib117] Warrant E.J., Nilsson D.-E. (1998). Absorption of white light in photoreceptors. Vision Res..

[bib118] Westerfield M. (2000). The Zebrafish Book. A Guide for the Laboratory Use of Zebrafish (Danio rerio).

[bib119] Wichmann C., Moser T. (2015). Relating structure and function of inner hair cell ribbon synapses. Cell Tissue Res..

[bib120] Wilson C. (2012). Aspects of larval rearing. ILAR J..

[bib121] Wood S.N. (2006). Generalized Additive Models: an Introduction with R.

[bib122] Xiao T., Baier H. (2007). Lamina-specific axonal projections in the zebrafish tectum require the type IV collagen Dragnet. Nat. Neurosci..

[bib123] Yau K.-W., Hardie R.C. (2009). Phototransduction motifs and variations. Cell.

[bib124] Yilmaz M., Meister M. (2013). Rapid innate defensive responses of mice to looming visual stimuli. Curr. Biol..

[bib125] Yoshimatsu T., Williams P.R.P.R., D’Orazi F.D.F.D., Suzuki S.C., Fadool J.M., Allison W.T.T., Raymond P.A.P.A., Wong R.O.R.O. (2014). Transmission from the dominant input shapes the stereotypic ratio of photoreceptor inputs onto horizontal cells. Nat. Commun..

[bib126] Yoshimatsu T., D’Orazi F.D., Gamlin C.R., Suzuki S.C., Suli A., Kimelman D., Raible D.W., Wong R.O. (2016). Presynaptic partner selection during retinal circuit reassembly varies with timing of neuronal regeneration in vivo. Nat. Commun..

[bib127] Zang J., Keim J., Kastenhuber E., Gesemann M., Neuhauss S.C.F. (2015). Recoverin depletion accelerates cone photoresponse recovery. Open Biol..

[bib128] Zhou M., Bear J., Roberts P., Janiak F., Semmelhack J., Yoshimatsu T., Baden T. (2020). What the zebrafish’s eye tells the zebrafish’s brain: retinal ganglion cells for prey capture and colour vision. bioRxiv.

[bib129] Zimmermann M.J.Y., Nevala N.E., Yoshimatsu T., Osorio D., Nilsson D.-E., Berens P., Baden T. (2018). Zebrafish differentially process color across visual space to match natural scenes. Curr. Biol..

[bib130] Zimmermann M.J.Y., Chagas A.M., Bartel P., Pop S., Godino L.L.P., Baden T. (2020). LED Zappelin’: an open source LED controller for arbitrary spectrum visual stimulation and optogenetics during 2-photon imaging. bioRxiv.

